# An Update on Zoonotic *Cryptosporidium* Species and Genotypes in Humans

**DOI:** 10.3390/ani11113307

**Published:** 2021-11-19

**Authors:** Una Ryan, Alireza Zahedi, Yaoyu Feng, Lihua Xiao

**Affiliations:** 1 Harry Butler Institute, Murdoch University, Perth, WA 6152, Australia; A.ZahediAbdi@murdoch.edu.au; 2 Center for Emerging and Zoonotic Diseases, College of Veterinary Medicine, South China Agricultural University, Guangzhou 510642, China; yyfeng@scau.edu.cn (Y.F.); lxiao1961@gmail.com (L.X.); 3 Guangdong Laboratory for Lingnan Modern Agriculture, Guangzhou 510642, China

**Keywords:** *Cryptosporidium*, zoonotic, transmission, molecular tools

## Abstract

**Simple Summary:**

*Cryptosporidium* is a parasite that infects humans and a broad range of animals. There are few diagnostic features that can be used to identify and differentiate between species and therefore DNA-based detection and genetic typing methods are required. This is important as some species are transmitted from animals to humans (a process called zoonotic transmission) and understanding this is central to control. These DNA-based tools have greatly facilitated our current understanding of which of the many *Cryptosporidium* species and genotypes that have been identified are capable of infecting humans. More studies need to be conducted in areas where the potential for zoonotic transmission is greatest and recently developed genetic tools should be applied more extensively to further our understanding of the zoonotic transmission of this important parasite.

**Abstract:**

The enteric parasite, *Cryptosporidium* is a major cause of diarrhoeal illness in humans and animals worldwide. No effective therapeutics or vaccines are available and therefore control is dependent on understanding transmission dynamics. The development of molecular detection and typing tools has resulted in the identification of a large number of cryptic species and genotypes and facilitated our understanding of their potential for zoonotic transmission. Of the 44 recognised *Cryptosporidium* species and >120 genotypes, 19 species, and four genotypes have been reported in humans with *C. hominis, C. parvum, C. meleagridis, C. canis* and *C. felis* being the most prevalent. The development of typing tools that are still lacking some zoonotic species and genotypes and more extensive molecular epidemiological studies in countries where the potential for transmission is highest are required to further our understanding of this important zoonotic pathogen. Similarly, whole-genome sequencing (WGS) and amplicon next-generation sequencing (NGS) are important for more accurately tracking transmission and understanding the mechanisms behind host specificity.

## 1. Introduction

*Cryptosporidium* species are enteric parasites with a global distribution and a wide range of hosts. Transmission is by the faecal–oral route via contaminated water, food or direct contact with humans and animals [1]. Although first described in 1907 by Tyzzer [2], *Cryptosporidium* did not come to prominence until the early 1980s, when it was identified as a cause of severe protracted diarrhoea and death in HIV+/AIDS patients [3]. It is now recognised as a major pathogen in children and immunocompromised adults [4,5] and after rotavirus is the most important diarrheal pathogen in young children [6,7]. In 1993, *Cryptosporidium* was responsible for a large waterborne outbreak affecting over 400,000 residents of Milwaukee, Wisconsin [8] and although still frequently under-reported, is a well-known and major cause of both waterborne and foodborne outbreaks of gastroenteritis globally [9,10,11]. This is due in part to the resistance of the environmental stage, the oocyst, to disinfectants including chlorine treatment of both drinking and recreational water [12,13].

The parasite has a complex life cycle that initiates upon ingestion and excystation of oocysts and involves both asexual and sexual phases, which culminate in the shedding of infectious thick-walled oocysts in faeces [14]. In humans, in addition to watery diarrhoea, cryptosporidiosis can cause abdominal pain, vomiting, headaches, joint pain, malnutrition, failure to thrive and cognitive deficits and has been linked with colon cancer [15,16,17,18,19,20]. Although usually self-limiting in immunocompetent hosts, cryptosporidiosis can become chronic, persisting for 2 years or more [5,17]. In neonatal livestock, cryptosporidiosis can cause profuse diarrhoea, weight loss and death [11,21,22,23], resulting in significant production losses [22,24,25,26]. The only US Food and Drug Administration approved drug, nitazoxanide, is ineffective in the most affected populations (children and immunocompromised individuals) and there is no FDA-approved vaccine [27,28,29,30]. In animals, nitazoxanide is also largely ineffective and while Halocur^®^ (halofuginone lactate) has been licensed in some countries as a prophylactic, its effectiveness is variable and it cannot be given to animals that already have diarrhoea [11,22].

Molecular typing of *Cryptosporidium* has advanced significantly over the last few decades and is particularly important for this parasite as many species are morphologically identical [31]. Identification to species level is most commonly conducted using the 18S ribosomal RNA locus due to the existence of both hypervariable and conserved sequences [31,32]. Other loci examined include actin, heat shock protein 70 (*hsp7*0) and the *Cryptosporidium* oocyst wall protein gene (COWP) [31]. Currently, at least 44 species of *Cryptosporidium* and >120 genotypes have been identified, with *C. hominis* and *C. parvum* being the most important species infecting humans [33].

Contact with animals has been identified as a risk factor in many studies [34,35,36,37]; therefore, zoonotic transmission plays a major role in the epidemiology of cryptosporidiosis [1,23,38,39,40,41]. In order to track transmission, multi-locus sequence typing (MLST) tools have been developed, including analysis of the highly polymorphic glycoprotein 60 (*gp60*) gene, the most commonly used locus [31]. The *gp60*-based typing system is based on a combination of tandem serine-coding trinucleotide repeats (TCA, TCG and TCT) and extensive sequence divergence in non-repeat regions [31,42,43]. The nomenclature system [43], starts with a Roman numeral and lower-case letter for each *Cryptosporidium* and genotype (e.g., *C. hominis*, Ia, Ib, etc., *C. parvum* IIa, IIb, etc.), followed by uppercase letters denoting numbers of repeats. For example, *C. hominis* subtype IfA19G1R5 has 19 TCA (A) repeats, 1 TCG repeat (G) repeat and 5 AAGAAGGCAAAGAAG repeats (R). For some divergent species and genotypes, including *C. ubiquitum, C. felis, C. canis, C. ryanae, C. xiaoi, C. bovis* and *Cryptosporidium* chipmunk genotype I and skunk genotype, whole-genome sequencing (WGS) has been required to identify the *gp60* loci in order to develop typing systems [44,45,46,47,48,49,50]. The trinucleotide repeats (TCA, TCG, TCT) are absent from *gp60* genes in *C. ubiquitum, C. felis, C. canis, C. ryanae, C. bovis* and *C. xiaoi* [44,46,47,48,49,50].

This review aims to summarise the currently available data on the zoonotic transmission of *Cryptosporidium* species and genotypes as well as outlining future studies that are required to better understand the transmission dynamics of this ubiquitous parasite. This review could provide a valuable reference for One Health scientists and help guide future research.

## 2. Zoonotic *Cryptosporidium* Species and Genotypes

**Figure 1:**
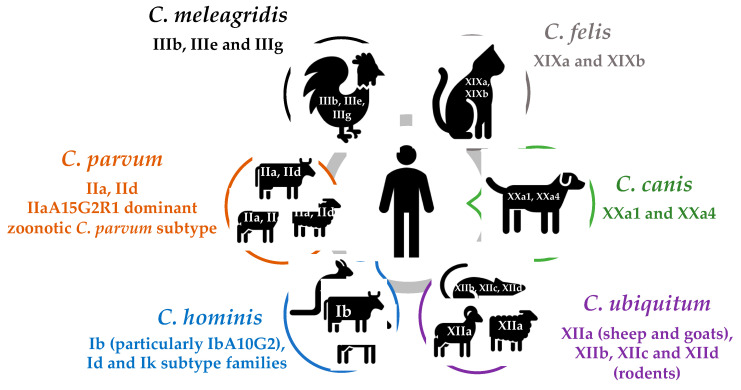
Zoonotic transmission of the six most common species of *Cryptosporidium* in humans.

In total, 19 species (including C. hominis and C. parvum) and 4 genotypes have been reported in humans *including C. meleagridis, C. canis, C. felis, C. ubiquitum, C. cuniculus, C. viatorum, C. muris, C. andersoni, C. erinacei, C. tyzzeri, C. bovis, C. suis, C. scrofarum, C. occultus, C. xiaoi, C. fayeri, C. ditrichi, Cryptosporidium* chipmunk genotype I, mink genotype, skunk genotype and horse genotype [1,33] (Table 1). Where possible, subtyping analysis at the gp60 locus has been used to document *gp60* subtypes common to humans and animals, to support zoonotic transmission (Table 2). Cryptosporidium hominis and C. parvum are responsible for ~95% of human infections, followed by *C. meleagridis, C. felis, C. canis* and *C. ubiquitum* [1,38] (Figure 1). Some species such as *C. meleagridis, C. canis, C. felis, C. viatorum* and *C. muris* are more commonly identified in humans in developing countries, whereas others such as *C. ubiquitum, C. cuniculus* and chipmunk genotype I are predominantly seen in developed countries [1].

### 2.1. Cryptosporidium hominis

*Cryptosporidium hominis* is the dominant species in humans in many industrialised countries and in developing countries, whereas, in the Middle East, European countries and New Zealand, *C. parvum* occurs at similar rates to *C. hominis* [1], indicating that zoonotic transmission is more prevalent in countries with intensive farming [122]. Although largely anthropologically transmitted, there have been numerous reports of *C. hominis* in animals (including non-human primates, cattle, sheep, goats, horses, donkeys, Bactrian camels, birds, marsupials, a dugong, badger, dingo, foxes, flying fox, rodents, and fish) and experimental infections has been established in calves, lambs, piglets, gerbils, and mice [123,124,125]. In human infectivity trials, the 50% infectious dose (ID_50_) was as low as 10 *C. hominis* oocysts [126], however, in animal models, much larger numbers of oocysts were required to achieve infections, suggesting that higher doses are required to cause infection in animals [125].

*Cryptosporidium hominis* has been detected at a relatively low frequency in livestock [22,23,125], although in one study, 80% of *Cryptosporidium* positives in calves in New Zealand were identified as *C. hominis* [127] and *C. hominis* was the only *Cryptosporidium* sp. detected in goats in South Korea [128]. These studies demonstrate that cattle, sheep and goats can potentially serve as animal reservoirs for infections with *C. hominis* [22,23,125]. In Australia, in addition to cattle and sheep, *C. hominis* has been detected in deer, dingoes and marsupials inhabiting drinking water catchments [129,130,131]. This likely resulted from human spill-back either via direct contact or via sewage contamination of pastures or drinking water.

The *C. hominis* subtypes identified at the *gp60* locus in non-human hosts has been extensively reviewed [125]. Briefly, the *C. hominis* IbA10G2 subtype dominates in livestock that are positive for *C. hominis* [22,125] and has also been detected in hedgehogs and mice in Europe [132,133,134]. As IbA10G2 is one of the most prevalent subtypes in humans in many countries and is also a major cause of waterborne outbreaks [1,11,38], this further supports a human origin for *C. hominis* in animals. In equines and particularly donkeys and horses, the Ik *C. hominis* subtype family (which is relatively rare in humans) is endemic and a reservoir of infection for humans [135,136,137,138]. Studies in Australia also suggest that *C. hominis* may become endemic in marsupials [131,139,140]. Continued human encroachment into wildlife areas may result in *C. hominis* becoming increasingly endemic in livestock and wildlife populations escalating the risk of zoonotic *C. hominis* transmission.

### 2.2. Cryptosporidium parvum and Other Livestock-Associated Species

*Cryptosporidium parvum* has a very wide host range infecting ungulates, wildlife (including carnivores, rodents, non-human primates, marine mammals and fish) and is the most important zoonotic species in humans, particularly in rural areas with frequent contact with livestock [1,11,22,23,124,141,142]. Calves, sheep and goats are major contributors to zoonotic *C. parvum* transmission with significant differences in prevalence [11,22,23,143]. In cattle, the dominant species are *C. parvum*, *C. bovis*, *C. ryanae* and *C. andersoni*, although other species (*C. hominis*, *C. felis*, *C. suis*, *C. scrofarum*, *C. meleagridis*, *C. tyzzeri*, *C. serpent* is, a novel genotype and *C. occultus*) have been reported [11,22]. In sheep and goats, *C. parvum*, *C. ubiquitum* and *C. xiaoi* are the predominant species, but a range of other species including *C. bovis*, *C. hominis*, *C. andersoni*, *C. suis*, *C. muris*, *C. fayeri*, *C. baileyi*, *C. ryanae*, *C. scrofarum*, *C. canis*, *C. occultus*, *Cryptosporidium* sheep genotype I and rat genotype II have occasionally been reported [11,22,23]. In pre-weaned calves, *C. parvum* is the dominant species in most studies [22], with the exception of China where *C. bovis* mostly dominates [144]. There does not appear to be an age-related prevalence of *C. parvum* in sheep and goats and while *C. parvum* dominates in these hosts in Europe and Australia, in other regions including the Americas, Asia, *C. xiaoi* and *C. ubiquitum* dominate in sheep and goats [23].

Over 20 *C. parvum gp60* subtype families have been identified with geographic variation in subtype distribution as well as host adaptation demonstrated [23,31]. For example, the three dominant *C. parvum gp60* subtype families in humans are IIa, IId and IIc, and of these, IIc appears to be almost exclusively anthropologically transmitted whereas IIa and IId are zoonotic [1,33,145]. WGS also supports this and it has been suggested that the *C. parvum* IIc subtype should be considered a separate subspecies (*Cryptosporidium parvum anthroponosum*), while the zoonotically transmitted IIa and IId subtypes referred to as *Cryptosporidium parvum parvum* [146]. In cattle, IIa is the dominant *C. parvum* subtype family in most countries and particularly in Europe and contact with calves has been identified as a risk factor for infection with the IIa subtypes [34]. In other countries, however, including China, Egypt, Malaysia, Romania and Sweden, the IId subtype dominates in cattle [31,144]. Similarly in sheep and goats, IIa dominates in European countries but in Africa, Asia and Australia, IId is the dominant *C. parvum* subtype family [23,144]. Subtyping studies have confirmed the identification of identical IIa and IId subtypes in humans and animals indicating zoonotic transmission [11,22,23,31,117,139]. For example, the relatively rare IIaA19G1R1 was identified in children, goat kids, and lambs during an outbreak of cryptosporidiosis at a holiday farm in Norway [147]. Similarly, subtypes IIaA15G2R1, IIaA18G2R1 and IIaA19G1R1 were responsible for several outbreaks in the UK, with the same subtypes identified from livestock on the same farms where the outbreaks occurred [38]. Of these, IIaA15G2R1 is the dominant *C. parvum* subtype in humans and cattle in industrialised countries and MLST analysis indicates that genetic recombination is the driving force behind its emergence as a dominant and hyper-transmissible subtype [148].

*Cryptosporidium bovis* is prevalent in cattle and other bovid species, while *C. xiaoi* is a related species in sheep and goats worldwide [11,22,23]. A *gp60* typing system has been established for *C. bovis* based on WGS sequences. Sixty-eight subtypes in six subtype families (XXVIa to XXVIf) have been identified, with apparent genetic recombination among subtype families [50]. There have been several reports of *C. bovis* in humans (Table 1). It was first reported in a dairy farm worker (and dairy calves) in Bengal, India in 2010 [108] and again in 2012 in a study in Australia, which collected and screened faecal samples from diarrheic calves in rural NSW and farmers from these farms [109]. In that study, *C. bovis* was identified in a 3-year-old child and a 23-year-old adult, from separate farms and both were asymptomatic [109]. Both individuals drank raw milk and had frequent contact with calves [109]. In another study, a mixed *C. parvum* and *C. bovis* infection was identified in a diarrhoeic child (<6 years) (along with mixed infections in cattle) from Ismailia province in Egypt [110]. A *gp60* typing system has recently been established for *C. xiaoi* based on WGS, with 12 subtype families, (XXIIIa to XXIIIl) and high subtype diversity identified [48]. It has previously been reported in two HIV/AIDS patients from Ethiopia based on 18S sequences only [34]. Both *C. bovis* and *C. xiaoi* appear to have narrow host ranges but further research is required to understand the zoonotic potential of these common livestock species.

Like *C. parvum*, *C. ubiquitum* (previously cervine genotype) also has a wide host range and is commonly detected in both domestic and wild ruminants, rodents, carnivores, primates, and humans, particularly in industrialised countries [44,51,52,139,149,150]. Both *gp60* and MLST tools have been developed for *C. ubiquitum* [44,150]. At the *gp60* locus, *C. ubiquitum* lacks the TCA, TCG and TCT repeats and to date, eight subtype families (XIIa–XIIh) have been identified [44], yet the transmission routes between animals and humans are not well understood. In the UK, XIIa is the dominant *C. ubiquitum* subtype in humans, which is also the main subtype in small ruminants [23,44]. In contrast, human *C. ubiquitum* infections in the US are caused by XIIb, XIIc and XIId subtype families which are common in rodents [44]. Subtype families XIIe and XIIf are also found in rodents but at present have only been identified in the Slovak Republic [44]. In France and Sweden, XIIb and XIId subtype families have been reported in humans [51,52]. MLST analysis has also re-affirmed that XIIa is ruminant-adapted and XIIb, XIIc and XIId subtype families are rodent-adapted [150].

*Cryptosporidium andersoni*, a gastric species, was originally thought to be *C. muris* due to its morphological similarity, until it was established as a separate species [151]. It is commonly reported in ruminants, particularly adult cattle and other bovids and is also common in sheep and goats, mainly in China [23] and has been reported in deer [152] and rodents, particularly hamsters [153,154,155,156]. As gastric *Cryptosporidium* species do not appear to have the *gp60* gene, a *gp60* typing tool has not been established for *C. andersoni*. However, an MLST tool has been developed and a mostly epidemic population structure has been identified [157,158,159]. There have been numerous reports in humans, but the extent of zoonotic transmission remains to be determined (Table 1).

*Cryptosporidium suis* and *C. scrofarum* are the dominant species infecting pigs (and wild boars) and mostly cause subclinical infections (although *C. parvum*, *C. muris*, *C. tyzzeri*, *C. felis*, *C. hominis*, *C. andersoni* and *C. meleagridis* can also infect pigs) [160,161,162]. *Cryptosporidium suis* is mainly found in pre-weaned animals and *C. scrofarum* in post-weaned pigs. Neither *C. suis* nor *C. scrofarum* are commonly identified in humans. There have been a few reports of *C. suis* in HIV-positive individuals in China, Peru and Thailand [79,92,101,102,103], as well as children in Cambodia [99] and symptomatic individuals in the UK and Madagascar [96,100]. There has only been one report of *C. scrofarum* in a symptomatic immunocompetent 29-year-old male co-infected with *Giardia* [121]. A *gp60* typing tool has not yet been developed for *C. suis* or *C. scrofarum* and need to be developed to explore the zoonotic potential of *C. suis* and *C. scrofarum* in pigs and transmission routes via water and sewage.

The horse genotype is commonly reported in donkeys and horses [135,137,138]. Typing of the horse genotype at the *gp60* locus has identified subtype families VIa, VIb and VIc [31,51]. Subtype VIaA15G4 is one of the most common subtypes identified in horses and donkeys, with subtype VIbA13 and VIaA14G4 identified in a hedgehog and calf respectively [135]. The horse genotype (subtype VIcA16) has been identified in a traveller to Sweden [51], an 18-year-old symptomatic patient from the US (subtype VIbA13) [104], a symptomatic 30-year-old immunocompetent woman from the rural UK (subtype VIbA13) [105] and in two adult females from the UK [82].

### 2.3. C. meleagridis

Avian cryptosporidiosis was first described in 1929 [163], but the first avian species, *Cryptosporidium meleagridis* was not named until 1955 in turkeys [164]. The parasite has a broad host range and is commonly reported in wild birds but less so in poultry [165,166]. It has also been reported in foxes, minks, cattle, wallabies, gorillas, and dogs [167,168,169,170,171,172]. Experimental transmission studies have also confirmed its infectivity for calves, pigs, rabbits, rats and mice and humans [173,174]. It is the third most common *Cryptosporidium* species in humans [1,175] and has also been identified in colon adenocarcinoma tissue in an immunocompetent Polish patient [176]. Phylogenetic analysis suggests that *C. meleagridis* may originally have been a mammalian parasite that secondarily became established in birds [177]. In children and HIV+ individuals in developing countries, *C. meleagridis* is frequently the dominant *Cryptosporidium* species [1,5,178], whereas, in developed countries, *C. meleagridis* is usually responsible for ~1–4% of human infections [38,66,174,179]. Despite being so commonly identified in humans, the extent of zoonotic transmission is not well understood. One study in an organic farm in Sweden identified identical *C. meleagridis* 70 kDa Heat Shock Protein (*hsp70*) gene sequences in samples from one human, three chickens and one hen, suggesting zoonotic transmission [180].

Typing at the *gp60* locus indicates that humans are susceptible to most *C. meleagridis gp60* subtypes, as of the 10 known subtype families (IIIa to IIIi), eight (IIIa to IIIh) (and 30 subtypes) have been identified in humans with IIIb among the most common [31,181,182,183,184]. Relatively few subtyping studies have been conducted in birds but both MLST and *gp60* studies have provided some support for zoonotic transmission. For example, subtype IIIgA31G3R1 has been reported from four poultry and one human from a Swedish farm [181]. MLST analysis of *C. meleagridis* from humans and birds from Peru did not find evidence of host segregation [182] and the same *gp60* subtypes (IIIbA26G1R1b and IIIbA22G1R1c) have been found in children with diarrhoea and in farmed chickens in Hubei province, China, supporting zoonotic transmission [183,184]. Similarly, subtypes IIIeA17G2R1, IIIeA19G2R1, IIIeA21G2R1 and IIIeA22G1R1 has been reported in Swedish patients (and IIIeA21G2R1 in Canadian patients) and in rodents and chickens in Asia [51,52,79,183] (Table 2).

### 2.4. Companion Animal-Associated Species (C. canis and C. felis)

Companion animals, particularly dogs and cats, have close relationships with their owners contributing to improved mental health and social support but can also contribute to zoonotic disease transmission [185]. There are currently ~470 million pet dogs and 370 million pet cats in the world (www.statista.com/statistics/1044386/dog-and-cat-pet-population-worldwide/accessed on 20 October 2021). *Cryptosporidium canis* and *C. felis* are the main species infecting dogs and cats respectively and are among the top five *Cryptosporidium* species infecting humans [142,186]. There have also been numerous reports of *C. parvum* and a few reports of *C. meleagridis*, *C. muris*, *C. andersoni*, *C. scrofarum*, *C. ryanae*, *C. hominis* and rat genotype III in cats and dogs, some of which may be due to coprophagy [40,187]. Prevalence rates of *C. canis* and *C. felis* vary widely but are commonly below 10% [40,187,188]. Few studies have examined the species of *Cryptosporidium* in humans and pets living in the same household, but one study identified *C. canis* in a 32-month-old girl, her 6.5-year-old brother and a dog from the same house in Lima, Peru [189]. Another study reported identical 18S, HSP70 and COWP *C. felis* sequences from a cat and her 37-year-old immunocompetent owner in Sweden [190]. A case-control study of HIV-infected individuals with and without cryptosporidiosis reported only a weak association between dog ownership and cryptosporidiosis [191].

*Cryptosporidium canis* and *C. felis gp60* loci have recently been characterised and have been shown to lack the serine-coding trinucleotide repeats normally used for typing (similar to *C. ubiquitum*) [40,46,49,186]. Within *C. canis*, five *gp60* subtype families have been identified (XXa, XXb, XXc, XXd and XXe), with subtypes XXa1 and XXa4 detected in both humans and dogs [49]. The previously identified household transmission of *C. canis* between two children and a dog in Lima, Peru [190] was confirmed by *gp60* subtyping [49]. Five subtype families have also been identified in *C. felis* (XIXa, XIXb, XIXc, XIXd and XIXe) [46] and of these, two subtypes (XIXa and XIXb) have been reported in both humans and cats supporting zoonotic transmission, with the remaining three subtypes (XIXc, XIXd and XIXe), possibly transmitted anthropologically [40,46,186,192]. 

### 2.5. Wildlife-Associated Species and Genotypes

Due to their wide geographical distribution and abundance and close contact with humans, rodents are considered an important zoonotic reservoir for *Cryptosporidium* and the global prevalence of *Cryptosporidium* in rodents has been estimated at ~17% [193]. To date, cryptosporidial infections from five rodent-derived species (*C. viatorum*, *C. muris*, *C. tyzzeri*, *C. occultus* and *C. ditrichi*) and one genotype (*Cryptosporidium* chipmunk genotype I) have been reported in humans (Table 1). Of these, there have only been a few reports of *C. tyzzeri*, *C. occultus* and *C. ditrichi* in humans (Table 1).

*Cryptosporidium muris*, a gastric species, has a very wide host range including rodents, ruminants, cats and dogs, horses, Bactrian camels, pigs, birds, and non-human primates [22,23,40,162,194,195,196,197,198]. There have been numerous reports in humans, particularly in developing countries (in children and HIV patients) [1,5], (Table 1). As with *C. andersoni*, an MLST tool has been developed for *C. muris* [157,158], which revealed that the genetic diversity of *C. muris* was greater than *C. andersoni*, possibly reflecting the much smaller host range of *C. andersoni* and that host clustering was evident, suggesting that some *C. muris* isolates have co-evolved with their hosts over a long period of time [158]. A *C. muris* human infectivity trial has been conducted in which six healthy adults were challenged with 10^5^ *C. muris* oocysts each (isolate RN66) and all became infected (two were symptomatic), confirming the zoonotic potential of *C. muris* [199].

Typing of *C. tyzzeri* is at the *gp60* locus has identified three subtype families; IXa, IXb and IXc [31,200], including subtype IXbA22R9 from a horse [195]. *Cryptosporidium tyzzeri* (subtype IXaA6R2) was identified from a child in Kuwait [43] and subtype family IXb has been detected in three human patients in New Zealand [54]. A mixed *C. parvum* (subtype IIaA13G1R1) and *C. tyzzeri* (subtypes IXaA8 and IXbA6) infection was identified in a symptomatic 25-year-old female conducting fieldwork trapping wild rodents in the Czech Republic [113]. The same subtypes were identified in the trapped mice supporting zoonotic transmission [113]. *Cryptosporidium tyzzeri* is genetically very closely related to *C. parvum* [201], which further supports its potential for zoonotic transmission.

A *gp60* typing system has not been established for *C. ditrichi* or *C. occultus*. *Cryptosporidium ditrichi* has been reported in three patients from Sweden, two of which were symptomatic and one had possible contact with mice [51,116]. Although predominantly a rodent species, *C. occultus* appears to be common in bovids including cattle, yaks (*Bos gunniens*) and water buffalo (*Bubalus bubalis*) [202] and was the dominant species detected in Alpacas in China [203]. It has been reported in one human from the UK (HQ822146), two humans from British Columbia [115] and a young child in China [114]. 

*Cryptosporidium viatorum* was originally reported in the UK in travellers returning from India and formally described as a human species in 2012 [73]. Since then, *C. viatorum* have been described in humans from Australia [66], China [114], Columbia [68], Ethiopia [34,64,74], India [63,67], Mozambique [62], Myanmar [65], Nigeria [69,70] and Swedish patients (who had returned from Kenya or Guatemala) [51,71,72]. It has also been detected in urban wastewater in China [204]. Due to the relatively high prevalence of *C. viatorum* in rats in Australia and China [205,206,207], it is now thought that *C. viatorum* was originally a rodent species and therefore rodents are likely to be an important reservoir host. 

A *gp60* typing tool has been established for *C. viatorum* and to date four subtype families have been identified; XVa, XVb, XVc and XVd [205,206,207,208]. Subtyping supports the potential zoonotic transmission between rodents and humans. For example, human-derived *C. viatorum* isolates have been subtyped to date as XVa3a to XVa3h [66,114,209] and XVcA2G1c [65], and subtype XVaA6 was identified in both sewer overflow and wastewater in Shanghai, China [204]. In rodents, XVa (XVaA6, XVaA3g, XVaA3h), XVb (XVbA2G1), XVc (XVcA2G1a, XVcA2G1b) and XVd (XVdA3) subtype families have been identified [205,206,207]. Thus, three subtypes (XVaA3g, XVaA3h and XVcA2G1) are common to humans and rodents and in addition, the XVaA3g subtype identified from wild rats [206] was 100% homologous to an XVaA3g subtype identified in a human patient in Australia [66]. Screening of rodents and rats across wider geographic areas is essential to better understand their role as reservoirs for *C. viatorum*.

To date, five chipmunk genotypes have been identified in rodents but only one of these, chipmunk genotype 1 has been identified in humans [33]. Chipmunk genotype I was first identified in New York storm water as genotype W17 [209] but was renamed as chipmunk genotype I, when it was detected in rodent faecal samples in 2007 [210]. It infects a range of rodents particularly squirrels, chipmunks, and deer mice and is considered an emerging zoonotic pathogen in humans [45,75], being the third most commonly identified *Cryptosporidium* sp. in Sweden [75]. A *gp60* typing system has been developed for chipmunk genotype I, with one subtype family identified (XIVa) [45]. Chipmunk genotype I was first identified in two humans in 2004 in Wisconsin (subtype XIVaA16G2T1) [77], and in a 41-year-old HIV-positive male in France [60]. In 2013, it was reported in a two-year-old female and 56-year-old male from Sweden [71,72] and typed as XIVaA20G2T1 by Guo et al. [45], who also identified 19 US human isolates and typed 17 as XIVaA14G2T1 (*n* = 1), XIVaA16G2T1 (*n* = 1), XIVaA14G2T2 (*n* = 1), XIVaA16G2T2 (*n* = 4), XIVaA18G2T1b (*n* = 2), XIVaA17G2T2 (*n* = 1), XIVaA19G2T2a (*n* = 1), XIVaA20G2T2 (*n* = 2), XIVaA19G2T2b (*n* = 1), XIVaA15G2T3 (*n* = 2) and XIVaA17G2T3 (*n* = 1) [45]. In that study, in the three rodent samples analysed, subtypes XIVaA18G2T1a and XIVaA18G2T2 were detected. It was identified in a human in Nebraska [37] and between 2014 and 2015, chipmunk genotype 1 was identified in five adults (four women and one man) in Sweden and all were typed as XIVaA20G2T1 [51]. Subsequently, it was identified in sixteen sporadic cases, three outbreak-related cases, and one zoonotic case, as well as in two squirrel samples in Sweden between 2018 and 2019, and subtyping of nineteen humans and two squirrels identified subtype XIVaA20G2T1 in all samples, supporting zoonotic transmission [75]. 

Of the other wildlife species and genotypes identified in humans, *C. cuniculus* (previously known as rabbit genotype) is a common species in rabbits worldwide [211,212,213] and has also been reported in kangaroos and alpacas in Australia [130,131,214]. It is the only species (other than *C. parvum* and *C. hominis*) known to have caused a waterborne outbreak of disease [56,61]. It is more commonly reported in humans in industrialised countries and particularly in the UK (Table 1). Two subtype families (Va and Vb) have been identified [31]. The waterborne outbreak caused by *C. cuniculus* in the UK was typed as VaA18 [56], however, most *C. cuniculus* cases typed in humans and rabbits have been caused by subtype family Vb [51,52,53,54,55,58]. In humans, subtypes VaA11, VaA18, VaA19 to VaA22, VaA23, VbA13, VbA15 VbA17, VbA20, VbA22, VbA23, VbA24 to VbA34, VbA36, VbA37 and VbA38 have been reported [51,52,54,58,211,212]. Of these, subtypes VbA19, VbA22 to Vb26, Vb28, VbA29 and VbA31 to VbA33 have been reported in rabbits supporting zoonotic transmission [130,131,140,212,215,216,217,218]. More recently an MLST typing tool has been developed and a clonal population structure identified [219]. 

*Cryptosporidium erinacei* (previously hedgehog genotype 1) was first described in European hedgehogs in 2014 [220]. It has also been reported in horses in Algeria and rats in China [206,221] and was detected in cattle and kangaroo faecal samples by next-generation amplicon sequencing in Australia [131]. *Cryptosporidium erinacei* has been reported in an immunocompetent 26-year-old man from the Czech Republic [107], an immunocompromised patient in France [106], in two cases from Sweden (one locally acquired and one in a traveller from Greece) [51] and in thirteen cases from New Zealand [53,54]. The higher number of cases in New Zealand may be due to the greater abundance of European hedgehogs (*Erinaceus europaeus*) in New Zealand compared to Europe, but as *C. erinacei* can infect other hosts, the range and prevalence of this parasite in reservoir hosts remain to be determined.

At the *gp60* locus, one subtype family (XIIIa) has been identified [31,220]. Zoonotic transmission has not been demonstrated as the *gp60* subtypes identified in humans (XIIIaA20R10, XIIIaA23R12, XIIIaA24R9, XIIIaA24R10, XIIIaA25R10 XIIIaA25R11, XIIIaA26R9 and XIIIaA26R10) [51,54] have not been identified in hedgehogs and horses (XIIIaA19R12, XIIIaA19R13, XIIIaA21R10, XIIIaA21R11, XIIIaA22R9, XIIIaA22R11 [221,222,223]). However as only a small number of isolates have been subtyped, further research is required to better understand its zoonotic potential.

The skunk genotype was first identified in striped skunks (*Mephitis mephitis*) in the US [177], but has a broad host range and has been described in numerous species of wild mammals [111,223], and is common in surface water in the US and Canada [111]. There have been five reports in humans; an 18-month-old child attending a UK daycare centre [112], a 25-year-old woman from a rural area in the UK [105], an adult female from the UK [82] and a human in Nebraska [37,111], and all except the child attending daycare were symptomatic.

Typing at the *gp60* locus has identified four subtype families (XVIa, XVIb, XVIc and XVId) with a high subtype diversity (*n* = 14) [111,224]. In humans, subtypes XVIbA16G2b and XVIcA22 have been identified, which have not been identified in wildlife. However, a closely related subtype (XVIbA16G2a subtype) has been found in raccoons and storm runoff samples in the US [111]. 

There have only been two reports of the marsupial derived *C. fayeri* in humans; a symptomatic 29-year-old immunocompetent female in New South Wales (NSW), Australia in 2010 and a decade later, in 2020, it was identified in a symptomatic 37-year-old immunosuppressed female treated for acute myeloid leukaemia in Western Australia (WA) [119,120]. Eight subtype families have been identified within *C. fayeri* (Iva–IVh) [31,130,225,226]. In the initial study by Waldron et al. [119], the same *C. fayeri* subtype (IVaA9G4T1R1) was identified in both the woman and eastern grey kangaroos (*Macropus giganteus*) inhabiting the main drinking water catchment for Sydney [225] and the woman had regular contact with marsupials supporting zoonotic transmission [119]. In the latter study in WA, *C. fayeri* subtype IVgA10G1T1R1 was identified in the female [120] and the same subtype had previously been identified in western grey kangaroos (*Macropus fuliginosus*) in WA and rabbits (*Oryctolagus cuniculus*) in NSW drinking water catchments [131], again supporting the potential for zoonotic transmission. Marsupials are the dominant animals inhabiting drinking water catchments in Australia and further research is necessary to better understand the zoonotic implications, particularly in addition to *C. fayeri* and *C. macropodum*, marsupials can also be infected with *C. hominis* as discussed above [131].

The *Cryptosporidium* mink genotype was first identified in minks (*Mustela vison*) inhabiting a New York City drinking water supply watershed [210] and is a common parasite in minks [227]. Typing at the *gp60* locus has identified four subtypes, including XaA5G1, XbA5G1R1, XcA5G1R1 and XdA4G1 [172,227,228]. There have been two reports of the mink genotype in humans [117,118]. However, no *gp60* sequences are available and therefore the possibility of zoonotic transmission is unknown.

**Table 2:** Zoonotic *gp60* subtypes common to humans and animals (excluding non-human primates).

Species Name	*gp60* Subtypes	References
*C. hominis*	IbA9G3, IbA13G3, IbA14G2, IbA10G2, IdA15G1, IbA10G2R2 and Ik subtype family	[22,125,127,131,132,133,134,139,221]
*C. parvum*	Many subtypes but mainly the IIa (particularly IIaA15G2R1) and IId subtype families	[11,22,23,31,117,140]
*C. meleagridis*	IIIbA21G1R1b, IIIbA22G1R1cIIIbA23G1R1b, IIIbA23G1R1c, IIIbA24G1R1, IIIbA26G1R1b, IIIeA17G2R1, IIIeA19G2R1, IIIeA21G2R1, IIIeA21G2R1 and IIIgA31G3R1	[51,52,79,181,182,183,184]
*C. felis*	XIXa and XIXb	[40,46,186,192]
*C. canis*	XXa1 and XXa4	[40,49]
*C. ubiquitum*	XIIa, XIIb, XIIc and XIId	[23,44,51,52]
*C. cuniculus*	VaA18, VbA19, VbA22 to Vb26, Vb28, VbA29 and VbA31 to VbA33	[51,52,53,54,55,56,58,210,211]
*C. viatorum*	XVaA3g, XVaA3h and XVcA2G1	[65,66,114,207,209]
Chipmunk genotype I	XIVaA18G2T2	[37,45,51,60,71,72,75,76,77]
*C. muris*	Gastric *Cryptosporidium* species do not appear to have the *gp60* gene	
*C. andersoni*		
*C. suis*	A *gp60* typing tool has not yet been developed	
Horse genotype	VIbA13 and VIcA16	[51,58,82,104,105]
*C. erinacei*	XIIIaA20R10, XIIIaA23R12, XIIIaA24R9, XIIIaA24R10, XIIIaA25R10 XIIIaA25R11, XIIIaA26R9 and XIIIaA26R10 (humans only)	[53,54]
*C. bovis*	No *gp60* sequences available from humans	
Skunk genotype	XVIbA16G2b and XVIcA22 (humans only but XVIbA16G2a in wildlife)	[111,224]
*C. tyzzeri*	IXaA8 and IXbA6	[43,113]
*C. occultus*	A *gp60* typing tool has not yet been developed	
*C. ditrichi*	A *gp60* typing tool has not yet been developed	
Mink genotype	No *gp60* sequences available from humans	
*C. fayeri*	IVaA9G4T1R1, IVgA10G1T1R1	[119,120]
*C. xiaoi*	No *gp60* sequences available from humans	
*C. scrofarum*	A *gp60* typing tool has not yet been developed	

## 3. Knowledge Gaps and Future Studies

The prevalence and clinical impact of *Cryptosporidium* and the potential for zoonotic transmission is highest in developing countries, yet relatively few molecular epidemiological studies have been conducted in these areas [1,33]. Large-scale molecular studies are therefore urgently required to explore the extent of zoonotic transmission [1,33]. Relatively little information is also available on the pathogenicity of some of the less common zoonotic species and genotypes, as most reports do not provide detailed clinical information, and this is another important knowledge gap that needs to be addressed. The majority of *gp60* subtyping studies conducted to date have relied on Sanger sequencing, which cannot reliably identify the extent of mixed subtypes. For example, a recent study applied the bioinformatic program TIDE to deconvolute *gp60* chromatograms generated using Sanger sequencing and identified previously unrecognised mixed subtype infections and has the advantage of also being able to be applied to retrospective data [229]. Similarly, another bioinformatics tool called “CryptoGenotyper” has been developed to read both 18S and *gp60* Sanger sequence data, which can also resolve double peaks in mixed infections and increase the accuracy of sequence identification [230]. Amplicon next-generation sequencing (NGS), which can identify low-abundance sequences in mixed infections, has shown that it can identify additional *Cryptosporidium gp60* subtypes not identified by Sanger sequencing in various hosts [141,231]. This has important implications for tracing zoonotic transmission as Sanger sequencing may not detect zoonotic species and subtypes that are present at low abundance and therefore incorrect conclusions regarding zoonotic transmission may be made. Future studies need to examine the extent of intra-sample diversity more closely to better understand zoonotic transmission dynamics.

The application of CRISPR-Cas (Clustered Regularly Interspaced Short Palindromic Repeats-CRISPR associated protein) genome editing technology to *Cryptosporidium* has been a major advance in the ability to genetically modify the parasite [232]. More recently CRISPR technologies have been applied to the detection of infectious diseases by identifying specific pathogen sequences and then cleaving them in order to produce a readable signal [233]. For example, in the HOLMES (one-HOur Low-cost Multipurpose highly Efficient System) detection platform, if a target DNA is present, a Cas protein called Cas12a forms a complex with the guide CRISPR RNA, which then binds the target DNA and cleaves a non-target ssDNA reporter in the system, resulting in a fluorescent signal [233]. A lateral flow biosensor has been developed which incorporated isothermal amplification of *Cryptosporidium* DNA with this CRISPR detection method to identify *C. parvum* sequences belonging to the *gp60* IId subtype family (from both humans and cattle), with high sensitivity and specificity [234]. This type of system has the potential to be employed in field situations to rapidly identify zoonotic subtypes and transmission hot-spots for more targeted analysis.

A more complete understanding of the molecular epidemiology of *Cryptosporidium* and zoonotic transmission dynamics can be gained from WGS studies, which are also increasingly being used to identify *gp60* loci in divergent species and genotypes [235]. Currently, WGS data has been generated from the following zoonotic species and genotypes; *C. parvum*, *C. hominis*, *C. muris*, *C. meleagridis*, *C. ubiquitum*, *C. andersoni*, *C. tyzzeri*, *C. cuniculus*, *C. viatorum*, *C. felis*, *C. canis*, *C. bovis*, *C. xiaoi*, *Cryptosporidium* skunk genotype and chipmunk genotype I [44,45,46,47,48,49,50,236]. Comparative WGS has identified a variety of invasion-associated proteins including mucin glycoproteins, insulinase-like proteases and MEDLE secretory proteins, which differ between species with narrow and broad host ranges and hence may play a role in host specificity [76,237,238,239]. However, many challenges remain including the development of a universal MLST system that uses polymorphic markers distributed across all chromosomes [236]. Future studies need to conduct WGS on a wider range of species and genotypes and to examine the biological functions of diverse genes in the *Cryptosporidium* genome to gain a better understanding of the genetic determinants of host specificity.

WGS is expensive and another alternative may be to mine existing raw untargeted (shotgun) sequencing metagenomic data deposited in public databases generated for other purposes (such as the microbiome of humans and animals) [240,241]. For example, one study successfully mined 12 large data sets on the human gut microbiome to examine the population genomics of *Blastocystis* [242]. As a proof of principle, *C. parvum* (chromosome 6) was identified and mapped from data sets derived from water concentrates and calf microbiomes, although further bioinformatic pipeline optimisation is required [243]. With the rapid expansion of publicly available metagenomes, this approach offers a cost-effective and unprecedented opportunity to unravel the extent of zoonotic transmission and environmental contamination of *Cryptosporidium* across wide geographic areas.

## 4. Conclusions

Our understanding of the extent of genetic diversity and zoonotic potential of *Cryptosporidium* species and genotypes has been greatly enhanced by the development of molecular detection and typing tools, particularly the *gp60* typing tool. The six most common species identified in humans are *C. hominis*, *C. parvum*, *C. meleagridis*, *C. canis*, *C. felis* and *C. ubiquitum*, yet the extent of zoonotic transmission in many of these species is still not clearly understood. Although primarily anthropologically transmitted, *C. hominis* is increasingly detected in animals, with evidence that it may be endemic in marsupials (IbA10G2 subtype) and equines (Ik subtype family). *Cryptosporidium parvum* is the most important zoonotic species, with IIa and IId the major zoonotic subtypes. Evidence to date supports the zoonotic transmission of *C. meleagridis* (IIIbA26G1R1b and IIIbA22G1R1c subtypes), *C. canis* (XXa1 and XXa4 subtypes), *C. felis* (XIXa and XIXb subtypes) and *C. ubiquitum* (XIIa, XIIb, XIIc and XIId subtype families). *Cryptosporidium viatorum* was originally described as a human species but rodents are now thought to be the major host species with subtypes (XVaA3g, XVaA3h and XVcA2G1) common to both humans and rodents. The rodent-derived chipmunk genotype I is considered an emerging human pathogen in the US and Sweden with evidence supporting the zoonotic transmission (subtype XIVaA20G2T1) between humans and squirrels. *Cryptosporidium cuniculus* in rabbits is another important zoonotic species, as it was responsible for a waterborne outbreak in the UK, with evidence supporting zoonotic transmission of multiple subtypes. *Cryptosporidium erinacei* in hedgehogs is a minor zoonotic species in most areas except New Zealand and the limited *gp60* subtyping conducted to date has yet to support zoonotic transmission. Similarly, identical skunk genotype *gp60* subtypes have not been identified in skunks and humans. Two studies have identified identical *C. fayeri* subtypes (IVaA9G4T1R1 and IVgA10G1T1R1) in humans and kangaroos in Australia. Gastric species (*C. muris* and *C. andersoni*) seem to lack the *gp60* gene but *gp60* typing tools that are still lacking for *C. suis*, *C. scrofarum*, *C. ditrichi*, *C. occultus* and the mink genotype are essential to better understand their transmission dynamics. The application of WGS and amplicon NGS in future studies are important for more accurately tracking transmission and understanding the mechanisms behind host specificity.

## Figures and Tables

**Table 1:** *Cryptosporidium* species and genotypes in humans in order of frequency.

Species Name	Main Reservoir Hosts	Reports in Humans
*C. hominis*	Non-human primates, donkeys	Most common species in humans
*C. parvum*	Ruminants, wildlife	Second most common species in humans
*C. meleagridis*	Birds	Third most commonly reported species in humans
*C. felis*	Cats	Commonly reported [40]
*C. canis* (previously canine genotype)	Dog	Commonly reported [40]
*C. ubiquitum* (previously cervine genotype)	Ruminants, rodents, carnivores, non-human primates	Commonly reported [31,44,51,52]
*C. cuniculus* (previously rabbit genotype)	Rabbits	Many reports in humans [51,52,53,54,55,56,57,58,59,60,61]
*C. viatorum*	Rodents	[34,51,62,63,64,65,66,67,68,69,70,71,72,73,74]
Chipmunk genotype I	Rodents	[37,45,51,60,71,72,75,76,77]
*C. muris*	Rodents	[52,60,78,79,80,81,82,83,84,85,86,87,88,89]
*C. andersoni* (previously *C. muris*-like genotype)	Cattle	[65,81,90,91,92,93,94,95,96,97]
*C. suis* (previously pig genotype I)	Pigs, wild boars	[79,96,98,99,100,101,102,103]
Horse genotype	Horses	[51,58,82,104,105]
*C. erinacei* (previously hedgehog genotype)	Hedgehogs, horses	[51,53,54,106,107]
*C. bovis* (previously bovine B genotype)	Cattle	[78,108,109,110]
Skunk genotype	Skunk	[37,82,105,111,112]
*C. tyzzeri* (previously mouse genotype I)	Rodents	[43,54,113]
*C. occultus* (previously *C. suis*-like genotype and *C. parvum* VF383)	Rodents	Refs. [114,115] Unpublished report in a human in GenBank (HQ822146)
*C. ditrichi*	Rodents (mainly mice)	[51,116]
Mink genotype	Mink	[117,118]
*C. fayeri* (previously marsupial genotype I)	Marsupials	[119,120]
*C. xiaoi* (previously *C. bovis*-like genotype)	Sheep, goats	[34]
*C. scrofarum* (previously pig genotype II)	Pigs, wild boar	[121]

## References

[1] Yang X., Guo Y., Xiao L., Feng Y. (2021). Molecular epidemiology of human cryptosporidiosis in low- and middle-income countries. Clin. Microbiol. Rev..

[2] Tyzzer E.E. (1907). A sporozoan found in the peptic glands of the common mouse. Proc. Soc. Exp. Biol. Med..

[3] Centers for Disease Control (1982). Cryptosporidiosis: Assessment of chemotherapy of males with acquired immune deficiency syndrome (AIDS). MMWR Morb. Mortal. Wkly. Rep..

[4] Shrivastava A., Kumar S., Smith W.A., Sahu P.S. (2017). Revisiting the global problem of cryptosporidiosis and recommendations. Trop. Parasitol..

[5] Wang R.-J., Li J.-Q., Chen Y.-C., Zhang L.-X., Xiao L.-H. (2018). Widespread occurrence of *Cryptosporidium* infections in patients with HIV/AIDS: Epidemiology, clinical feature, diagnosis, and therapy. Acta Trop..

[6] Kotloff K.L., Nataro J.P., Blackwelder W.C., Nasrin D., Farag T.H., Panchalingam S., Wu Y., Sow S.O., Sur D., Breiman R.F. (2013). Burden and aetiology of diarrhoeal disease in infants and young children in developing countries (the Global Enteric Multicenter Study, GEMS): A prospective, case-control study. Lancet.

[7] Levine M.M., Nasrin D., Ac’acio S., Bassat Q., Powell H., Tennant S.M., Sow S.O., Sur D., Zaidi A.K., Faruque A.S.G. (2020). Diarrhoeal disease and subsequent risk of death in infants and children residing in low-income and middle-income countries: Analysis of the GEMS case-control study and 12-month GEMS-1A follow-on study. Lancet Glob. Health..

[8] Mac Kenzie W.R., Schell W.L., Blair K.A., Addiss D.G., Peterson D.E., Hoxie N.J., Kazmierczak J.J., Davis J.P. (1995). Massive Outbreak of Waterborne *Cryptosporidium* Infection in Milwaukee, Wisconsin: Recurrence of Illness and Risk of Secondary Transmission. Clin. Infect. Dis..

[9] Efstratiou A., Ongerth J.E., Karanis P. (2017). Waterborne transmission of protozoan parasites: Review of worldwide outbreaks—An update 2011–2016. Water Res..

[10] Ryan U., Hijjawi N., Xiao L. (2018). Foodborne cryptosporidiosis. Int. J. Parasitol..

[11] Zahedi A., Ryan U. (2020). *Cryptosporidium*—An update with an emphasis on foodborne and waterborne transmission. Res. Vet. Sci..

[12] King B., Monis P.T. (2007). Critical processes affecting *Cryptosporidium* oocyst survival in the environment. Parasitology.

[13] Gharpure R., Perez A., Miller A.D., Wikswo M.E., Silver R., Hlavsa M.C. (2019). Cryptosporidiosis outbreaks—United States, 2009–2017. MMWR Morb. Mortal Wkly. Rep..

[14] Gunasekera S., Zahedi A., O’Dea M., King B., Monis P., Thierry B., Carr J.M., Ryan U. (2020). Organoids and bioengineered intestinal models: Potential solutions to the *Cryptosporidium* culturing dilemma. Microorganisms.

[15] Guerrant D.I., Lima A., Patrick P.D., Schorling J.B., Moore S., Guerrant R.L. (1999). Association of early childhood diarrhea and cryptosporidiosis with impaired physical fitness and cognitive function four-seven years later in a poor urban community in northeast Brazil. Am. J. Trop. Med. Hyg..

[16] Mondal D., Sack R.B., Haque R., Jr W.A.P., Kirkpatrick B.D. (2009). Attribution of malnutrition to cause-specific diarrheal illness: Evidence from a prospective study of preschool children in Mirpur, Dhaka, Bangladesh. Am. J. Trop. Med. Hyg..

[17] Carter B.L., Chalmers R.M., Davies A.P. (2020). Health sequelae of human cryptosporidiosis in industrialised countries: A systematic review. Parasites Vectors.

[18] Kalantari N., Gorgani-Firouzjaee T., Ghaffari S., Bayani M., Ghaffari T., Chehrazi M. (2020). Association between *Cryptosporidium* infection and cancer: A systematic review and meta-analysis. Parasitol. Int..

[19] Pinto D.J., Vinayak S. (2021). *Cryptosporidium*: Host-Parasite Interactions and Pathogenesis. Curr. Clin. Microbiol. Rep..

[20] Sawant M., Baydoun M., Creusy C., Chabé M., Viscogliosi E., Certad G., Benamrouz-Vanneste S. (2020). *Cryptosporidium* and colon cancer: Cause or consequence?. Microorganisms.

[21] Thomson S., Hamilton C.A., Hope J.C., Katzer F., Mabbott N.A., Morrison L.J., Innes E.A. (2017). Bovine cryptosporidiosis: Impact, host-parasite interaction and control strategies. Vet. Res..

[22] Santin M. (2020). *Cryptosporidium* and *Giardia* in Ruminants. Vet. Clin. N. Am. Food Anim. Pract..

[23] Guo Y., Li N., Ryan U., Feng Y., Xiao L. (2021). Small ruminants and zoonotic cryptosporidiosis. Parasitol. Res..

[24] Jacobson C., Williams A., Yang R., Ryan U., Carmichael I., Campbell A.J., Gardner G. (2016). Greater intensity and frequency of *Cryptosporidium* and *Giardia* oocyst shedding beyond the neonatal period is associated with reductions in growth, carcase weight and dressing efficiency in sheep. Vet. Parasitol..

[25] Jacobson C., Al-Habsi K., Ryan U., Williams A., Anderson F., Yang R., Abraham S., Miller D. (2018). *Cryptosporidium* infection is associated with reduced growth and diarrhoea in goats beyond weaning. Vet. Parasitol..

[26] Shaw H.J., Innes E.A., Morrison L.J., Katzer F., Wells B. (2020). Long-term production effects of clinical cryptosporidiosis in neonatal calves. Int. J. Parasitol..

[27] Sears C.L., Kirkpatrick B.D. (2007). Is nitazoxanide an effective treatment for patients with acquired immune deficiency syndrome-related cryptosporidiosis?. Nat. Clin. Pract. Gastroenterol. Hepatol..

[28] Amadi B., Mwiya M., Sianongo S., Payne L., Watuka A., Katubulushi M., Kelly P. (2009). High dose prolonged treatment with nitazoxanide is not effective for cryptosporidiosis in HIV positive Zambian children: A randomised controlled trial. BMC Infect. Dis..

[29] Ashigbie P.G., Shepherd S., Steiner K.L., Amadi B., Aziz N., Manjunatha U.H., Spector J.M., Diagana T.T., Kelly P. (2021). Use-case scenarios for an anti-*Cryptosporidium* therapeutic. PLoS Negl. Trop. Dis..

[30] Crawford C.K., Kol A. (2021). The Mucosal Innate Immune Response to *Cryptosporidium parvum*, a Global one health issue. Front. Cell. Infect. Microbiol..

[31] Xiao L., Feng Y. (2017). Molecular epidemiologic tools for waterborne pathogens *Cryptosporidium* spp. and *Giardia duodenalis*. Food Waterborne Parasitol..

[32] Roellig D.M., Xiao L. (2020). *Cryptosporidium* Genotyping for epidemiology tracking. Methods Mol. Biol..

[33] Ryan U.M., Feng Y., Fayer R., Xiao L. (2021). Taxonomy and molecular epidemiology of *Cryptosporidium* and *Giardia*—A 50 year perspective (1971–2021). Int. J. Parasitol..

[34] Adamu H., Petros B., Zhang G., Kassa H., Amer S., Ye J., Feng Y., Xiao L. (2014). Distribution and clinical manifestations of *Cryptosporidium* species and subtypes in HIV/AIDS patients in Ethiopia. PLoS Negl. Trop. Dis..

[35] Bouzid M., Kintz E., Hunter P. (2018). Risk factors for *Cryptosporidium* infection in low and middle income countries: A systematic review and meta-analysis. PLoS Negl. Trop. Dis..

[36] Khan A., Shams S., Khan S., Khan M.I., Khan S., Ali A. (2019). Evaluation of prevalence and risk factors associated with *Cryptosporidium* infection in rural population of district Buner, Pakistan. PLoS ONE.

[37] Loeck B.K., Pedati C., Iwen P.C., McCutchen E., Roellig D.M., Hlavsa M.C., Fullerton K., Safranek T., Carlson A.V. (2020). Genotyping and subtyping *Cryptosporidium* to identify risk factors and transmission patterns—Nebraska, 2015–2017. MMWR Morb. Mortal. Wkly. Rep..

[38] Chalmers R.M., Robinson G., Elwin K., Elson R. (2019). Analysis of the *Cryptosporidium* spp. and gp60 subtypes linked to human outbreaks of cryptosporidiosis in England and Wales, 2009 to 2017. Parasites Vectors.

[39] Thomas-Lopez D., Müller L., Vestergaard L.S., Christoffersen M., Andersen A.-M., Jokelainen P., Agerholm J.S., Stensvold C.R. (2020). Veterinary students have a higher risk of contracting cryptosporidiosis when calves with high fecal *Cryptosporidium* loads are used for fetotomy exercises. Appl. Environ. Microbiol..

[40] Li J., Ryan U., Guo Y., Feng Y., Xiao L. (2021). Advances in molecular epidemiology of cryptosporidiosis in dogs and cats. Int. J. Parasitol..

[41] Smith R.P., Newton K., Rimdap E., Wight A., Robinson G., Chalmers R.M. (2021). Review of investigations of premises housing animals that were linked to human outbreaks of cryptosporidiosis in England and Wales between 2009 and 2019. Vet. Rec..

[42] Strong W.B., Gut J., Nelson R.G. (2000). Cloning and sequence analysis of a highly polymorphic *Cryptosporidium parvum* gene encoding a 60-kilodalton glycoprotein and characterization of Its 15- and 45-kilodalton zoite surface antigen products. Infect. Immun..

[43] Sulaiman I.M., Hira P.R., Zhou L., Al-Ali F.M., Al-Shelahi F.A., Shweiki H.M., Iqbal J., Khalid N., Xiao L. (2005). Unique endemicity of cryptosporidiosis in children in Kuwait. J. Clin. Microbiol..

[44] Li N., Xiao L., Alderisio K., Elwin K., Cebelinski E., Chalmers R., Santín M., Fayer R., Kvac M., Ryan U. (2014). Subtyping *Cryptosporidium ubiquitum*, A zoonotic pathogen emerging in humans. Emerg. Infect. Dis..

[45] Guo Y., Cebelinski E., Matusevich C., Alderisio K.A., Lebbad M., McEvoy J., Roellig D.M., Yang C., Feng Y., Xiao L. (2015). Subtyping novel zoonotic pathogen *Cryptosporidium* chipmunk genotype I. J. Clin. Microbiol..

[46] Rojas-López L., Elwin K., Chalmers R.M., Enemark H.L., Beser J., Troell K. (2020). Development of a *gp60*-subtyping method for *Cryptosporidium felis*. Parasites Vectors.

[47] Yang X., Huang N., Jiang W., Wang X., Li N., Guo Y., Kváč M., Feng Y., Xiao L. (2020). Subtyping *Cryptosporidium ryanae*: A Common pathogen in bovine animals. Microorganisms.

[48] Fan Y., Huang X., Guo S., Yang F., Yang X., Guo Y., Feng Y., Xiao L., Li N. (2021). Subtyping *Cryptosporidium xiaoi*, a common pathogen in sheep and goats. Pathogens.

[49] Jiang W., Roellig D.M., Guo Y., Li N., Feng Y., Xiao L. (2021). Development of a subtyping tool for zoonotic pathogen *Cryptosporidium canis*. J. Clin. Microbiol..

[50] Wang W., Wan M., Yang F., Li N., Xiao L., Feng Y., Guo Y. (2021). Development and Application of a *gp60*-Based Subtyping Tool for *Cryptosporidium bovis*. Microorganisms.

[51] Lebbad M., Winiecka-Krusnell J., Stensvold C., Beser J. (2021). High Diversity of *Cryptosporidium* Species and subtypes identified in cryptosporidiosis acquired in sweden and abroad. Pathogens.

[52] Guy R.A., Yanta C.A., Muchaal P.K., Rankin M.A., Thivierge K., Lau R., Boggild A.K. (2021). Molecular characterization of *Cryptosporidium* isolates from humans in Ontario, Canada. Parasites Vectors.

[53] Garcia–R J.C., French N., Pita A., Velathanthiri N., Shrestha R., Hayman D. (2017). Local and global genetic diversity of protozoan parasites: Spatial distribution of *Cryptosporidium* and *Giardia* genotypes. PLoS Negl. Trop. Dis..

[54] Garcia–R J.C., Pita A.B., Velathanthiri N., French N.P., Hayman D.T.S. (2020). Species and genotypes causing human cryptosporidiosis in New Zealand. Parasitol. Res..

[55] Koehler A.V., Whipp M.J., Haydon S.R., Gasser R.B. (2014). *Cryptosporidium cuniculus*—New records in human and kangaroo in Australia. Parasites Vectors.

[56] Puleston R.L., Mallaghan C.M., Modha D.E., Hunter P., Nguyen-Van-Tam J., Regan C.M., Nichols G.L., Chalmers R.M. (2014). The first recorded outbreak of cryptosporidiosis due to *Cryptosporidium cuniculus* (formerly rabbit genotype), following a water quality incident. J. Water Health.

[57] Martínez-Ruiz R., de Lucio A., Fuentes I., Carmena D. (2016). Autochthonous *Cryptosporidium cuniculus* infection in Spain: First report in a symptomatic paediatric patient from Madrid. Enferm. Infecc. Microbiol. Clin..

[58] Chalmers R.M., Elwin K., Hadfield S.J., Robinson G. (2011). Sporadic human cryptosporidiosis caused by *Cryptosporidium cuniculus*, United Kingdom, 2007–2008. Emerg. Infect. Dis..

[59] Molloy S.F., Kirwan P., Asaolu S.O., Holland C.V., Nichols R.A.B., Connelly L., Smith H.V. (2010). Identification of a High Diversity of *Cryptosporidium* Species Genotypes and Subtypes in a Pediatric Population in Nigeria. Am. J. Trop. Med. Hyg..

[60] Anofel *Cryptosporidium* National Network (2010). Laboratory-based surveillance for *Cryptosporidium* in France, 2006–2009. Eurosurveilliance.

[61] Chalmers R.M., Robinson G., Elwin K., Hadfield S.J., Xiao L., Ryan U., Modha D., Mallaghan C. (2009). *Cryptosporidium* sp. Rabbit genotype, a newly identified human pathogen. Emerg. Infect. Dis..

[62] Muadica A.S., Köster P.C., Dashti A., Bailo B., Hernández-de-Mingo M., Balasegaram S., Carmena D. (2021). Molecular diversity of *Giardia duodenalis*, *Cryptosporidium* spp. and *Blastocystis* sp. in symptomatic and asymptomatic school children in Zambezia province (Mozambique). Pathogens.

[63] Sardar S.K., Ghosal A., Saito-Nakano Y., Dutta S., Nozaki T., Ganguly S. (2021). Molecular Identification of *Cryptosporidium viatorum* infection in a patient suffering from unusual cryptosporidiosis in West Bengal, India. Korean J. Parasitol..

[64] Tarekegn Z.S., Tigabu Y., Dejene H. (2021). *Cryptosporidium* infection in cattle and humans in Ethiopia: A systematic review and meta-analysis. Parasite Epidemiol. Control.

[65] Wu Y., Gong B., Liu X., Jiang Y., Cao J., Yao L., Li H., Liu A., Shen Y. (2020). Identification of uncommon *Cryptosporidium viatorum* (a novel subtype XVcA2G1c) and *Cryptosporidium andersoni* as well as common *Giardia duodenalis* assemblages A and B in humans in Myanmar. Front. Cell. Infect. Microbiol..

[66] Braima K., Zahedi A., Oskam C., Reid S., Pingault N., Xiao L., Ryan U. (2019). Retrospective analysis of *Cryptosporidium* species in Western Australian human populations (2015–2018), and emergence of the *C*. hominis IfA12G1R5 subtype. Infect. Genet. Evol..

[67] Mirdha B.R., Khalil S., Paul J., Panda A., Singh Y. (2018). Molecular Detection and Identification of *Cryptosporidium viatorum* in a Human Immunodeficiency Virus-seropositive Patient. J. Glob. Infect. Dis..

[68] Sánchez A., Munoz M., Gómez N., Tabares J., Segura L., Salazar Á., Restrepo C., Ruíz M., Reyes P., Qian Y. (2017). Molecular Epidemiology of *Giardia*, *Blastocystis* and *Cryptosporidium* among Indigenous Children from the Colombian Amazon Basin. Front. Microbiol..

[69] Ukwah B.N., Ezeonu I.M., Ezeonu C.T., Roellig D., Xiao L. (2017). *Cryptosporidium* species and subtypes in diarrheal children and HIV-infected persons in Ebonyi and Nsukka, Nigeria. J. Infect. Dev. Ctries..

[70] Ayinmode A.B., Zhang H., Dada-Adegbola H.O., Xiao L. (2014). *Cryptosporidium hominis* Subtypes and *Enterocytozoon bieneusi* Genotypes in HIV-Infected Persons in Ibadan, Nigeria. Zoonoses Public Health.

[71] Lebbad M., Beser J., Insulander M., Karlsson L., Mattsson J.G., Svenungsson B., Axen C. (2013). Unusual cryptosporidiosis cases in Swedish patients: Extended molecular characterization of *Cryptosporidium viatorum* and *Cryptosporidium* chipmunk genotype *I*. J. Parasitol..

[72] Insulander M., Silverlås C., Lebbad M., Karlsson L., Mattsson J.G., Svenungsson B. (2013). Molecular epidemiology and clinical manifestations of human cryptosporidiosis in Sweden. Epidemiol. Infect..

[73] Elwin K., Hadfield S.J., Robinson G., Crouch N.D., Chalmers R.M. (2012). *Cryptosporidium viatorum* n. sp. (Apicomplexa: Cryptosporidiidae) among travellers returning to Great Britain from the Indian subcontinent, 2007–2011. Int. J. Parasitol..

[74] De Lucio A., Amor-Aramendía A., Bailo B., Saugar J.M., Anegagrie M., Arroyo A., López-Quintana B., Zewdie D., Ayehubizu Z., Yizengaw E. (2016). Prevalence and Genetic Diversity of *Giardia duodenalis* and *Cryptosporidium* spp. among School Children in a Rural Area of the Amhara Region, North-West Ethiopia. PLoS ONE.

[75] Bujila I., Troell K., Fischerström K., Nordahl M., Killander G., Hansen A., Söderlund R., Lebbad M., Beser J. (2021). *Cryptosporidium* chipmunk genotype I—An emerging cause of human cryptosporidiosis in Sweden. Infect. Genet. Evol..

[76] Xu Z., Guo Y., Roellig D.M., Feng Y., Xiao L. (2019). Comparative analysis reveals conservation in genome organization among intestinal *Cryptosporidium* species and sequence divergence in potential secreted pathogenesis determinants among major human-infecting species. BMC Genom..

[77] Feltus D.C., Giddings C.W., Schneck B.L., Monson T., Warshauer D., McEvoy J.M. (2006). Evidence supporting zoonotic transmission of *Cryptosporidium* spp. in Wisconsin. J. Clin. Microbiol..

[78] Higuera A., Villamizar X., Herrera G., Giraldo J.C., Vasquez-A. L.R., Urbano P., Villalobos O., Tovar C., Ramírez J.D. (2020). Molecular detection and genotyping of intestinal protozoa from different biogeographical regions of Colombia. PeerJ.

[79] Sannella A.R., Suputtamongkol Y., Wongsawat E., Cacciò S.M. (2019). A retrospective molecular study of *Cryptosporidium* species and genotypes in HIV-infected patients from Thailand. Parasites Vectors.

[80] Ayinmode A.B., Oliveira B.C.M., Obebe O.O., Dada-Adgebola H.O., Ayede A.I., Widmer G., Dada-Adegbola H. (2018). Genotypic Characterization of *Cryptosporidium* Species in Humans and Peri-Domestic Animals in Ekiti and Oyo States, Nigeria. J. Parasitol..

[81] Kattula D., Jeyavelu N., Prabhakaran A.D., Premkumar P.S., Velusamy V., Venugopal S., Geetha J.C., Lazarus R.P., Das P., Nithyanandhan K. (2017). Natural History of Cryptosporidiosis in a Birth Cohort in Southern India. Clin. Infect. Dis..

[82] Elwin K., Hadfield S.J., Robinson G., Chalmers R.M. (2011). The epidemiology of sporadic human infections with unusual cryptosporidia detected during routine typing in England and Wales, 2000–2008. Epidemiol. Infect..

[83] Al-Brikan F.A., Salem H.S., Beeching N., Hilal N. (2008). Multilocus genetic analysis of *Cryptosporidium* isolates from Saudi Arabia. J. Egypt. Soc. Parasitol..

[84] Gatei W., Kamwati S.K., Mbae C., Waruru A., Hart C.A., Wamae C.N., Revathi G., Mulinge E., Gatika S.M., Waithera T. (2006). Cryptosporidiosis: Prevalence, genotype analysis, and symptoms associated with infections in children in Kenya. Am. J. Trop. Med. Hyg..

[85] Muthusamy D., Rao S.S., Ramani S., Monica B., Banerjee I., Abraham O.C., Mathai D.C., Primrose B., Muliyil J., Wanke C.A. (2006). Multilocus Genotyping of *Cryptosporidium* sp. Isolates from Human immunodeficiency virus-infected individuals in South India. J. Clin. Microbiol..

[86] Palmer C.J., Xiao L., Terashima A., Guerra H., Gotuzzo E., Saldias G., Bonilla J.A., Zhou L., Lindquist A., Upton S.J. (2003). *Cryptosporidium muris*, a rodent pathogen, recovered from a human in Peru. Emerg. Infect. Dis..

[87] Gatei W., Ashford R.W., Beeching N.J., Kamwati S.K., Greensill J., Hart C.A. (2002). *Cryptosporidium muris* infection in an HIV-infected adult, Kenya. Emerg. Infect. Dis..

[88] Tiangtip R., Jongwutiwes S. (2002). Molecular analysis of *Cryptosporidium* species isolated from HIV-infected patients in Thailand. Trop. Med. Int. Health.

[89] Katsumata T., Hosea D., Uga S., Kohno S., Ranuh I.G., Yanagi T. (2000). Short report: Possible *Cryptosporidium muris* infection in humans. Am. J. Trop. Med. Hyg..

[90] Hussain G., Roychoudhury S., Singha B., Paul J. (2017). Incidence of *Cryptosporidium andersoni* in diarrheal patients from southern Assam, India: A molecular approach. Eur. J. Clin. Microbiol. Infect. Dis..

[91] Jiang Y., Ren J., Yuan Z., Liu A., Zhao H., Liu H., Chu L., Pan W., Cao J., Lin Y. (2014). *Cryptosporidium andersoni* as a novel predominant *Cryptosporidium* species in outpatients with diarrhea in Jiangsu Province, China. BMC Infect. Dis..

[92] Liu H., Shen Y., Yin J., Yuan Z., Jiang Y., Xu Y., Pan W., Hu Y., Cao J. (2014). Prevalence and genetic characterization of *Cryptosporidium*, *Enterocytozoon*, *Giardia* and *Cyclospora* in diarrheal outpatients in china. BMC Infect. Dis..

[93] Agholi M., Hatam G.M., Motazedian M.H. (2013). HIV/AIDS-associated opportunistic protozoal diarrhoea. AIDS Res. Hum. Retrovir..

[94] Waldron L.S., Dimeski B., Beggs P., Ferrari B., Power M.L. (2011). Molecular Epidemiology, Spatiotemporal Analysis, and Ecology of Sporadic Human Cryptosporidiosis in Australia. Appl. Environ. Microbiol..

[95] Morse T.D., Nichols R.A.B., Grimason A.M., Campbell B.M., Tembo K.C., Smith H.V. (2007). Incidence of cryptosporidiosis species in paediatric patients in Malawi. Epidemiol. Infect..

[96] Leoni F., Amar C., Nichols G., Pedraza-Díaz S., McLauchlin J. (2006). Genetic analysis of *Cryptosporidium* from 2414 humans with diarrhoea in England between 1985 and 2000. J. Med. Microbiol..

[97] Guyot K., Follet-Dumoulin A., Lelièvre E., Sarfati C., Rabodonirina M., Nevez G., Cailliez J.C., Camus D., Dei-Cas E. (2001). Molecular Characterization of *Cryptosporidium* Isolates Obtained from Humans in France. J. Clin. Microbiol..

[98] Liu A., Gong B., Liu X., Shen Y., Wu Y., Zhang W., Cao J. (2020). A retrospective epidemiological analysis of human *Cryptosporidium* infection in China during the past three decades (1987–2018). PLoS Negl. Trop. Dis..

[99] Moore C.E., Elwin K., Phot N., Seng C., Mao S., Suy K., Kumar V., Nader J., Bousfield R., Perera S. (2016). Molecular Characterization of *Cryptosporidium* Species and *Giardia duodenalis* from Symptomatic Cambodian Children. PLoS Negl. Trop. Dis..

[100] Bodager J.R., Parsons M.B., Wright P.C., Rasambainarivo F., Roellig D., Xiao L., Gillespie T.R. (2015). Complex epidemiology and zoonotic potential for *Cryptosporidium* suis in rural Madagascar. Vet. Parasitol..

[101] Wang L., Zhang H.-W., Zhao X., Zhang L., Zhang G., Guo M., Liu L., Feng Y., Xiao L. (2012). Zoonotic *Cryptosporidium* Species and *Enterocytozoon bieneusi* Genotypes in HIV-positive patients on antiretroviral therapy. J. Clin. Microbiol..

[102] Cama V.A., Ross J., Crawford S., Kawai V., Chavez-Valdez R., Vargas-Pacherrez D., Vivar A., Ticona E., Ñavincopa M., Williamson J. (2007). Differences in Clinical Manifestations among *Cryptosporidium* Species and Subtypes in HIV-Infected Persons. J. Infect. Dis..

[103] Xiao L., Bern C., Arrowood M., Sulaiman I., Zhou L., Kawai V., Vivar A., Lal A.A., Gilman R.H. (2002). Identification of the *Cryptosporidium* Pig genotype in a human patient. J. Infect. Dis..

[104] Xiao L., Hlavsa M.C., Yoder J., Ewers C., Dearen T., Yang W., Nett R., Harris S., Brend S.M., Harris M. (2009). Subtype analysis of *Cryptosporidium* specimens from sporadic cases in Colorado, Idaho, New Mexico, and Iowa in 2007: Widespread Occurrence of One *Cryptosporidium hominis* subtype and case history of an infection with the *Cryptosporidium* Horse Genotype. J. Clin. Microbiol..

[105] Robinson G., Elwin K., Chalmers R.M. (2008). Unusual *Cryptosporidium* genotypes in human cases of diarrhea. Emerg. Infect. Dis..

[106] Costa D., Razakandrainibe R., Sautour M., Valot S., Basmaciyan L., Gargala G., Lemeteil D., Favennec L., Dalle F., Debourgogne A. (2018). Human cryptosporidiosis in immunodeficient patients in France (2015–2017). Exp. Parasitol..

[107] Kvac M., Saková K., Květoňová D., Kicia M., Wesołowska M., McEvoy J., Sak B. (2014). Gastroenteritis Caused by the *Cryptosporidium* Hedgehog Genotype in an Immunocompetent Man. J. Clin. Microbiol..

[108] Khan S.M., Debnath C., Pramanik A.K., Xiao L., Nozaki T., Ganguly S. (2010). Molecular characterization and assessment of zoonotic transmission of *Cryptosporidium* from dairy cattle in West Bengal, India. Vet. Parasitol..

[109] Ng J.S.Y., Eastwood K., Walker B., Durrheim D.N., Massey P.D., Porigneaux P., Kemp R., McKinnon B., Laurie K., Miller D. (2012). Evidence of *Cryptosporidium* transmission between cattle and humans in northern New South Wales. Exp. Parasitol..

[110] Helmy Y.A., Krücken J., Nöckler K., von Samson-Himmelstjerna G., Zessin K.-H. (2013). Molecular epidemiology of *Cryptosporidium* in livestock animals and humans in the Ismailia province of Egypt. Vet. Parasitol..

[111] Yan W., Alderisio K., Roellig D.M., Elwin K., Chalmers R.M., Yang F., Wang Y., Feng Y., Xiao L. (2017). Subtype analysis of zoonotic pathogen *Cryptosporidium* skunk genotype. Infect. Genet. Evol..

[112] Davies A.P., Campbell B., Evans M.R., Bone A., Roche A., Chalmers R.M. (2009). Asymptomatic carriage of protozoan parasites in children in day care centers in the United Kingdom. Pediatr. Infect. Dis. J..

[113] Rasková V., Kvetonová D., Sak B., McEvoy J., Edwinson A., Stenger B., Kvác M. (2013). Human cryptosporidiosis is caused by *Cryptosporidium tyzzeri* and *C. parvum* isolates presumably transmitted from wild mice. J. Clin. Microbiol..

[114] Xu N., Liu H., Jiang Y., Yin J., Yuan Z., Shen Y., Cao J. (2020). First report of *Cryptosporidium viatorum* and *Cryptosporidium occultus* in humans in China, and of the unique novel *C. viatorum *subtype XVaA3h. BMC Infect. Dis..

[115] Ong C.S.L., Eisler D.L., Alikhani A., Fung V.W.K., Tomblin J., Bowie W.R., Isaac-Renton J.L. (2002). Novel *Cryptosporidium* genotypes in sporadic cryptosporidiosis cases: First report of human infections with a cervine genotype. Emerg. Infect. Dis..

[116] Beser J., Bujila I., Wittesjö B., Lebbad M. (2020). From mice to men: Three cases of human infection with *Cryptosporidium ditrichi*. Infect. Genet. Evol..

[117] Ng-Hublin J.S.Y., Combs B., MacKenzie B., Ryan U. (2013). Human Cryptosporidiosis Diagnosed in Western Australia: A Mixed Infection with *Cryptosporidium meleagridis*, the *Cryptosporidium* Mink Genotype, and an Unknown *Cryptosporidium* Species. J. Clin. Microbiol..

[118] Ebner J., Koehler A., Robertson G., Bradbury R., Jex A., Haydon S., Stevens M.A., Norton R., Joachim A., Gasser R.B. (2015). Genetic analysis of *Giardia* and *Cryptosporidium* from people in Northern Australia using PCR-based tools. Infect. Genet. Evol..

[119] Waldron L.S., Cheung-Kwok-Sang C., Power M.L. (2010). Wildlife-associated *Cryptosporidium fayeri* in human, Australia. Emerg. Infect. Dis..

[120] Braima K., Zahedi A., Oskam C., Austen J., Egan S., Reid S., Ryan U. (2021). Zoonotic infection by *Cryptosporidium fayeri* IVgA10G1T1R1 in a Western Australian human. Zoonoses Public Health.

[121] Kváč M., Květoňová D., Sak B., Ditrich O. (2009). *Cryptosporidium* Pig genotype II in immunocompetent man. Emerg. Infect. Dis..

[122] Robertson L.J., Johansen H., Kifleyohannes T., Efunshile A.M., Terefe G. (2020). *Cryptosporidium* Infections in Africa—How important is zoonotic transmission? A review of the evidence. Front. Vet. Sci..

[123] Barrera J.P., Carmena D., Rodríguez E., Checa R., López A.M., Fidalgo L.E., Gálvez R., Marino V., Fuentes I., Miró G. (2020). The red fox (*Vulpes vulpes*) as a potential natural reservoir of human cryptosporidiosis by *Cryptosporidium hominis* in Northwest Spain. Transbound. Emerg. Dis..

[124] Golomazou E., Malandrakis E.E., Panagiotaki P., Karanis P. (2021). *Cryptosporidium* in fish: Implications for aquaculture and beyond. Water Res..

[125] Widmer G., Köster P.C., Carmena D. (2020). *Cryptosporidium hominis* infections in non-human animal species: Revisiting the concept of host specificity. Int. J. Parasitol..

[126] Chappell C.L., Tzipori S., Akiyoshi D.E., Okhuysen P., Tanriverdi S., Langer-Curry R., Widmer G. (2006). *Cryptosporidium hominis*: Experimental challenge of healthy adults. Am. J. Trop. Med. Hyg..

[127] Abeywardena H., Jex A.R., Nolan M.J., Haydon S.R., Stevens M.A., McAnulty R.W., Gasser R.B. (2012). Genetic characterisation of *Cryptosporidium* and *Giardia* from dairy calves: Discovery of species/genotypes consistent with those found in humans. Infect. Genet. Evol..

[128] Park J.-H., Guk S.-M., Han E.-T., Shin E.-H., Kim J.-L., Chai J.-Y. (2006). Genotype analysis of *Cryptosporidium* spp. prevalent in a rural village in Hwasun-gun, Republic of Korea. Korean J. Parasitol..

[129] Ng J., Yang R., Whiffin V., Cox P., Ryan U. (2011). Identification of zoonotic *Cryptosporidium* and *Giardia* genotypes infecting animals in Sydney’s water catchments. Exp. Parasitol..

[130] Koehler A.V., Haydon S., Jex A.R., Gasser R.B. (2016). *Cryptosporidium* and *Giardia* taxa in faecal samples from animals in catchments supplying the city of Melbourne with drinking water (2011 to 2015). Parasites Vectors.

[131] Zahedi A., Monis P., Gofton A., Oskam C.L., Ball A., Bath A., Bartkow M., Robertson I., Ryan U. (2018). *Cryptosporidium* species and subtypes in animals inhabiting drinking water catchments in three states across Australia. Water Res..

[132] Krawczyk A.I., Van Leeuwen A.D., Jacobs-Reitsma W., Wijnands L.M., Bouw E., Jahfari S., Hoek A.H.A.M.V., Van Der Giessen J.W.B., Roelfsema J.H., Kroes M. (2015). Presence of zoonotic agents in engorged ticks and hedgehog faeces from *Erinaceus europaeus* in (sub) urban areas. Parasites Vectors.

[133] Danišová O., Valenčáková A., Stanko M., Luptáková L., Hatalová E., Canády A. (2017). Rodents as a reservoir of infection caused by multiple zoonotic species genotypes of *C*. *parvum*, *C*. *hominis*, *C. suis*, *C. scrofarum*, and the first evidence of *C. muskrat* genotypes I and II of rodents in Europe. Acta Trop..

[134] Condlová Š., Horčičková M., Sak B., Květoňová D., Hlásková L., Konecny R., Stanko M., McEvoy J., Kváč M. (2018). *Cryptosporidium* apodemi sp. n. and *Cryptosporidium ditrichi* sp. n. (Apicomplexa: Cryptosporidiidae) in *Apodemus* spp.. Eur. J. Protistol..

[135] Jian F., Liu A., Wang R., Zhang S., Qi M., Zhao W., Shi Y., Wang J., Wei J., Zhang L. (2016). Common occurrence of *Cryptosporidium hominis* in horses and donkeys. Infect. Genet. Evol..

[136] Inácio S.V., Widmer G., De Brito R.L.L., Zucatto A.S., De Aquino M.C.C., Oliveira B.C.M., Nakamura A.A., Neto L.D.S., Carvalho J.G.B., Gomes J.F. (2017). First description of *Cryptosporidium hominis* GP60 genotype IkA20G1 and *Cryptosporidium parvum* GP60 genotypes IIaA18G3R1 and IIaA15G2R1 in foals in Brazil. Vet. Parasitol..

[137] Li F., Su J., Chahan B., Guo Q., Wang T., Yu Z., Guo Y., Li N., Feng Y., Xiao L. (2019). Different distribution of *Cryptosporidium* species between horses and donkeys. Infect. Genet. Evol..

[138] Wang W., Zhang Z., Zhang Y., Zhao A., Jing B., Zhang L., Liu P., Qi M., Zhao W. (2020). Prevalence and genotypic identification of *Cryptosporidium* in free-ranging and farm-raised donkeys (*Equus asinus asinus*) in Xinjiang, China. Parasite.

[139] Zahedi A., Monis P., Aucote S., King B., Paparini A., Jian F., Yang R., Oskam C., Ball A., Robertson I. (2016). Zoonotic *Cryptosporidium* species in animals inhabiting sydney water catchments. PLoS ONE.

[140] Zahedi A., Gofton A.W., Jian F., Paparini A., Oskam C., Ball A., Robertson I., Ryan U. (2017). Next Generation Sequencing uncovers within-host differences in the genetic diversity of *Cryptosporidium* gp60 subtypes. Int. J. Parasitol..

[141] Zahedi A., Paparini A., Jian F., Robertson I., Ryan U. (2016). Public health significance of zoonotic *Cryptosporidium* species in wildlife: Critical insights into better drinking water management. Int. J. Parasitol. Parasites Wildl..

[142] Feng Y., Ryan U.M., Xiao L. (2018). Genetic diversity and population structure of *Cryptosporidium*. Trends Parasitol..

[143] Hatam-Nahavandi K., Ahmadpour E., Carmena D., Spotin A., Bangoura B., Xiao L. (2019). *Cryptosporidium* infections in terrestrial ungulates with focus on livestock: A systematic review and meta-analysis. Parasites Vectors.

[144] Feng Y., Xiao L. (2017). Molecular Epidemiology of Cryptosporidiosis in China. Front. Microbiol..

[145] King P., Tyler K.M., Hunter P.R. (2019). Anthroponotic transmission of *Cryptosporidium parvum* predominates in countries with poorer sanitation: A systematic review and meta-analysis. Parasites Vectors.

[146] Nader J.L., Mathers T., Ward B.J., Pachebat J., Swain M.T., Robinson G., Chalmers R.M., Hunter P.R., Van Oosterhout C., Tyler K.M. (2019). Evolutionary genomics of anthroponosis in *Cryptosporidium*. Nat. Microbiol..

[147] Lange H., Johansen O.H., Vold L., Robertson L.J., Anthonisen I.L., Nygard K. (2014). Second outbreak of infection with a rare *Cryptosporidium parvum* genotype in schoolchildren associated with contact with lambs/goat kids at a holiday farm in Norway. Epidemiol. Infect..

[148] Feng Y., Torres E., Li N., Wang L., Bowman D., Xiao L. (2013). Population genetic characterisation of dominant *Cryptosporidium parvum* subtype IIaA15G2R1. Int. J. Parasitol..

[149] Fayer R., Santín M., Macarisin D. (2010). *Cryptosporidium ubiquitum* n. sp. in animals and humans. Vet. Parasitol..

[150] Tang Y., Li N., Song M., Roellig D.M., Feng Y., Xiao L. (2016). Development of a multilocus sequence typing tool for high-resolution subtyping and genetic structure characterization of *Cryptosporidium ubiquitum*. Infect. Genet. Evol..

[151] Lindsay D.S., Upton S.J., Owens D.S., Morgan U.M., Mead J.R., Blagburn B.L. (2000). *Cryptosporidium andersoni* n. sp. (Apicomplexa: Cryptosporiidae) from Cattle, Bos taurus. J. Eukaryot. Microbiol..

[152] Huang J., Zhang Z., Zhang Y., Yang Y., Zhao J., Wang R., Jian F., Ning C., Zhang W., Zhang L. (2018). Prevalence and molecular characterization of *Cryptosporidium* spp. and *Giardia duodenalis* in deer in Henan and Jilin, China. Parasites Vectors.

[153] Chen S., Chai Y., Deng L., Liu H., Zhong Z., Fu H., Hu Y., Shen L., Zhou Z., Geng Y. (2021). *Cryptosporidium* spp. in Pet Dwarf Winter White Russian Hamsters (*Phodopus sungoris sungoris*) in China. J. Parasitol..

[154] Chen J., Wang W., Lin Y., Sun L., Li N., Guo Y., Kvac M., Ryan U., Feng Y., Xiao L. (2021). Genetic characterizations of *Cryptosporidium* spp. from pet rodents indicate high zoonotic potential of pathogens from chinchillas. One Health.

[155] Ježková J., Prediger J., Holubová N., Sak B., Konečný R., Feng Y., Xiao L., Rost M., McEvoy J., Kváč M. (2021). *Cryptosporidium* ratti n. sp. (Apicomplexa: Cryptosporidiidae) and genetic diversity of *Cryptosporidium* spp. in brown rats (*Rattus norvegicus*) in the Czech Republic. Parasitology.

[156] Lv C., Zhang L., Wang R., Jian F., Zhang S., Ning C., Wang H., Feng C., Wang X., Ren X. (2009). *Cryptosporidium* spp. in Wild, Laboratory, and Pet Rodents in China: Prevalence and molecular characterization. Appl. Environ. Microbiol..

[157] Feng Y., Yang W., Ryan U., Zhang L., Kváč M., Koudela B., Modrý D., Li N., Fayer R., Xiao L. (2011). Development of a Multilocus Sequence Tool for Typing *Cryptosporidium muris* and *Cryptosporidium andersoni*. J. Clin. Microbiol..

[158] Wang R., Jian F., Zhang L., Ning C., Liu A., Zhao J., Feng Y., Qi M., Wang H., Lv C. (2012). Multilocus sequence subtyping and genetic structure of *Cryptosporidium muris* and *Cryptosporidium andersoni*. PLoS ONE.

[159] Zhao W., Wang R., Zhang W., Liu A., Cao J., Shen Y., Yang F., Zhang L. (2014). MLST Subtypes and Population Genetic Structure of *Cryptosporidium andersoni* from dairy cattle and beef cattle in northeastern china’s heilongjiang province. PLoS ONE.

[160] Kváč M., Hanzlíková D., Sak B., Květoňová D. (2009). Prevalence and age-related infection of *Cryptosporidium suis*, *C*. *muris* and *Cryptosporidium* pig genotype II in pigs on a farm complex in the Czech Republic. Vet. Parasitol..

[161] García-Presedo I., Pedraza-Díaz S., González-Warleta M., Mezo M., Gómez-Bautista M., Ortega-Mora L.-M., Castro-Hermida J.A. (2013). Presence of *Cryptosporidium scrofarum*, *C. suis* and *C*. *parvum* subtypes IIaA16G2R1 and IIaA13G1R1 in Eurasian wild boars (*Sus scrofa*). Vet Parasitol..

[162] Ryan U., Fayer R., Xiao L. (2014). *Cryptosporidium* species in humans and animals: Current understanding and research needs. Parasitology.

[163] Tyzzer E.E. (1929). Coccidiosis in gallinaceous birds. Am. J. Epidemiol..

[164] Slavin D. (1955). *Cryptosporidium Meleagridis* (Sp. Nov.). J. Comp. Pathol. Ther..

[165] Baroudi D., Khelef D., Goucem R., Adjou K.T., Adamu H., Zhang H., Xiao L. (2013). Common occurrence of zoonotic pathogen *Cryptosporidium meleagridis* in broiler chickens and turkeys in Algeria. Vet. Parasitol..

[166] Wang Y., Zhang K., Chen Y., Li X., Zhang L. (2021). *Cryptosporidium* and cryptosporidiosis in wild birds: A One Health perspective. Parasitol. Res..

[167] Hajdušek O., Ditrich O., Šlapeta J. (2004). Molecular identification of *Cryptosporidium* spp. in animal and human hosts from the Czech Republic. Vet. Parasitol..

[168] Sak B., Petrželková K.J., Květoňová D., Mynářová A., Pomajbíková K.J., Modrý D., Cranfield M.R., Mudakikwa A., Kváč M. (2014). Diversity of Microsporidia, *Cryptosporidium* and *Giardia* in Mountain Gorillas (*Gorilla beringei beringei*) in Volcanoes National Park, Rwanda. PLoS ONE.

[169] Vermeulen E.T., Ashworth D.L., Eldridge M., Power M. (2015). Diversity of *Cryptosporidium* in brush-tailed rock-wallabies (*Petrogale penicillata*) managed within a species recovery programme. Int. J. Parasitol. Parasites Wildl..

[170] Zhang W., Wang R., Yang F., Zhang L., Cao J., Zhang X., Ling H., Liu A., Shen Y. (2013). Distribution and genetic characterizations of *Cryptosporidium* spp. in pre-weaned dairy calves in northeastern China’s Heilongjiang Province. PLoS ONE.

[171] Zhang S., Tao W., Liu C., Jiang Y., Wan Q., Li Q., Yang H., Lin Y., Li W. (2016). First report of *Cryptosporidium canis* in foxes (*Vulpes vulpes*) and raccoon dogs (*Nyctereutes procyonoides*) and identification of several novel subtype families for *Cryptosporidium* mink genotype in minks (*Mustela vison*) in China. Infect. Genet. Evol..

[172] Yang Z., Zhao W., Wang J., Ren G., Zhang W., Liu A. (2018). Molecular detection and genetic characterizations of *Cryptosporidium* spp. in farmed foxes, minks, and raccoon dogs in northeastern China. Parasitol. Res..

[173] Akiyoshi D.E., Dilo J., Pearson C., Chapman S., Tumwine J., Tzipori S. (2003). Characterization of *Cryptosporidium meleagridis* of Human Origin Passaged through Different Host Species. Infect. Immun..

[174] Chappell C.L., Okhuysen P., Tzipori S., Widmer G., Akiyoshi D.E., Langer-Curry R.C. (2011). *Cryptosporidium meleagridis*: Infectivity in Healthy Adult Volunteers. Am. J. Trop. Med. Hyg..

[175] Omolabi K.F., Odeniran P.O., Soliman M.E. (2021). A meta-analysis of *Cryptosporidium* species in humans from southern Africa (2000–2020). J. Parasit. Dis..

[176] Kopacz Ż., Kváč M., Karpiński P., Hendrich A.B., Sąsiadek M.M., Leszczyński P., Sak B., McEvoy J., Kicia M. (2019). The first evidence of *Cryptosporidium meleagridis* infection in a colon adenocarcinoma from an immunocompetent patient. Front. Cell Infect. Microbiol..

[177] Xiao L., Sulaiman I.M., Ryan U., Zhou L., Atwill E.R., Tischler M.L., Zhang X., Fayer R., Lal A.A. (2002). Host adaptation and host–parasite co-evolution in *Cryptosporidium*: Implications for taxonomy and public health. Int. J. Parasitol..

[178] Korpe P.S., Gilchrist C., Burkey C., Taniuchi M., Ahmed E., Madan V., Castillo R., Ahmed S., Arju T., Alam M. (2019). Case-Control Study of *Cryptosporidium* Transmission in Bangladeshi Households. Clin. Infect. Dis..

[179] Ng-Hublin J.S., Combs B., Reid S., Ryan U. (2017). Differences in the occurrence and epidemiology of cryptosporidiosis in Aboriginal and non-Aboriginal people in Western Australia (2002–2012). Infect. Genet. Evol..

[180] Silverlås C., Mattsson J.G., Insulander M., Lebbad M. (2012). Zoonotic transmission of *Cryptosporidium meleagridis* on an organic Swedish farm. Int. J. Parasitol..

[181] Stensvold C.R., Beser J., Axén C., Lebbad M. (2014). High Applicability of a Novel Method for gp60-Based Subtyping of *Cryptosporidium meleagridis*. J. Clin. Microbiol..

[182] Wang Y., Yang W., Cama V., Wang L., Cabrera L., Ortega Y., Bern C., Feng Y., Gilman R., Xiao L. (2014). Population genetics of *Cryptosporidium meleagridis* in humans and birds: Evidence for cross-species transmission. Int. J. Parasitol..

[183] Wang T., Fan Y., Koehler A., Ma G., Li T., Hu M., Gasser R.B. (2017). First survey of *Cryptosporidium*, *Giardia* and *Enterocytozoon* in diarrhoeic children from Wuhan, China. Infect. Genet. Evol..

[184] Liao C., Wang T., Koehler A.V., Fan Y., Hu M., Gasser R.B. (2018). Molecular investigation of *Cryptosporidium* in farmed chickens in Hubei Province, China, identifies ‘zoonotic’ subtypes of C. meleagridis. Parasites Vectors.

[185] Esch K.J., Petersen C.A. (2013). Transmission and epidemiology of zoonotic protozoal diseases of companion animals. Clin. Microbiol. Rev..

[186] Li J., Yang F., Liang R., Guo S., Guo Y., Li N., Feng Y., Xiao L. (2021). Subtype Characterization and Zoonotic Potential of *Cryptosporidium felis* in Cats in Guangdong and Shanghai, China. Pathogens.

[187] Lucio-Forster A., Griffiths J.K., Cama V.A., Xiao L., Bowman D.D. (2010). Minimal zoonotic risk of cryptosporidiosis from pet dogs and cats. Trends Parasitol..

[188] Taghipour A., Olfatifar M., Bahadory S., Godfrey S.S., Abdoli A., Khatami A., Javanmard E., Shahrivar F. (2020). The global prevalence of *Cryptosporidium* infection in dogs: A systematic review and meta-analysis. Vet. Parasitol..

[189] Xiao L., Cama V.A., Cabrera L., Ortega Y., Pearson J., Gilman R.H. (2007). Possible Transmission of *Cryptosporidium canis* among Children and a Dog in a Household. J. Clin. Microbiol..

[190] Beser J., Toresson L., Eitrem R., Troell K., Winiecka-Krusnell J., Lebbad M. (2015). Possible zoonotic transmission of *Cryptosporidium felis* in a household. Infect. Ecol. Epidemiol..

[191] Glaser C.A., Safrin S., Reingold A., Newman T.B. (1998). Association between *Cryptosporidium* infection and animal exposure in HIV-infected individuals. J. Acquir. Immune Defic. Syndr. Hum. Retrovirol..

[192] Jiang W., Roellig D.M., Lebbad M., Beser J., Troell K., Guo Y., Li N., Xiao L., Feng Y. (2020). Subtype distribution of zoonotic pathogen *Cryptosporidium felis* in humans and animals in several countries. Emerg. Microbes Infect..

[193] Taghipour A., Olfatifar M., Foroutan M., Bahadory S., Malih N., Norouzi M. (2020). Global prevalence of *Cryptosporidium* infection in rodents: A systematic review and meta-analysis. Prev. Vet. Med..

[194] Qi M., Huang L., Wang R., Xiao L., Xu L., Li J., Zhang L. (2014). Natural infection of *Cryptosporidium muris* in ostriches (*Struthio camelus*). Vet. Parasitol..

[195] Wagnerová P., Sak B., McEvoy J., Rost M., Matysiak A.P., Ježková J., Kváč M. (2015). Genetic diversity of *Cryptosporidium* spp. including novel identification of the *Cryptosporidium muris* and *Cryptosporidium tyzzeri* in horses in the Czech Republic and Poland. Parasitol. Res..

[196] Chen L., Hu S., Jiang W., Zhao J., Li N., Guo Y., Liao C., Han Q., Feng Y., Xiao L. (2019). *Cryptosporidium parvum* and *Cryptosporidium hominis* subtypes in crab-eating macaques. Parasites Vectors.

[197] García-Livia K., Martín-Alonso A., Foronda P. (2020). Diversity of *Cryptosporidium* spp. in wild rodents from the Canary Islands, Spain. Parasites Vectors.

[198] Wang L., Cao L., Zheng S., Chang Y., Zhang K., Zhang S., Zhang L. (2021). Molecular identification and biological characterization of *Cryptosporidium muris* from camels (*Camelus bactrianus*) in China. Parasites Vectors.

[199] Chappell C.L., Okhuysen P., Tzipori S., Widmer G., Lupo P., Langer-Curry R.C. (2015). *Cryptosporidium muris*: Infectivity and Illness in Healthy Adult Volunteers. Am. J. Trop. Med. Hyg..

[200] Čondlová Š., Horčičková M., Havrdová N., Sak B., Hlásková L., Perec-Matysiak A., Kicia M., McEvoy J., Kváč M. (2019). Diversity of *Cryptosporidium* spp. in *Apodemus* spp. in Europe. Eur. J. Protistol..

[201] Ren X., Zhao J., Zhang L., Ning C., Jian F., Wang R., Lv C., Wang Q., Arrowood M.J., Xiao L. (2012). *Cryptosporidium tyzzeri* n. sp. (Apicomplexa: Cryptosporidiidae) in domestic mice (*Mus musculus*). Exp. Parasitol..

[202] Kváč M., Vlnatá G., Ježková J., Horčičková M., Konečný R., Hlásková L., McEvoy J., Sak B. (2018). *Cryptosporidium occultus* sp. n. (Apicomplexa: Cryptosporidiidae) in rats. Eur. J. Protistol..

[203] Zhang Q., Li J., Li Z., Xu C., Hou M., Qi M. (2020). Molecular identification of *Cryptosporidium* spp. in alpacas (*Vicugna pacos*) in China. Int. J. Parasitol. Parasites Wildl..

[204] Huang C., Hu Y., Wang L., Wang Y., Li N., Guo Y., Feng Y., Xiao L. (2017). Environmental Transport of Emerging Human-Pathogenic *Cryptosporidium* Species and Subtypes through Combined Sewer Overflow and Wastewater. Appl. Environ. Microbiol..

[205] Koehler A.V., Wang T., Haydon S.R., Gasser R.B. (2018). *Cryptosporidium viatorum* from the native Australian swamp rat Rattus lutreolus-An emerging zoonotic pathogen?. Int. J. Parasitol. Parasites Wildl..

[206] Chen Y.-W., Zheng W.-B., Zhang N.-Z., Gui B.-Z., Lv Q.-Y., Yan J.-Q., Zhao Q., Liu G.-H. (2019). Identification of *Cryptosporidium viatorum* XVa subtype family in two wild rat species in China. Parasites Vectors.

[207] Zhao W., Zhou H., Huang Y., Xu L., Rao L., Wang S., Wang W., Yi Y., Zhou X., Wu Y. (2019). *Cryptosporidium* spp. in wild rats (*Rattus* spp.) from the Hainan Province, China: Molecular detection, species/genotype identification and implications for public health. Int. J. Parasitol. Parasites Wildl..

[208] Stensvold C.R., Elwin K., Winiecka-Krusnell J., Chalmers R.M., Xiao L., Lebbad M. (2015). Development and Application of a gp60-Based Typing Assay for *Cryptosporidium viatorum*. J. Clin. Microbiol..

[209] Jiang J., Alderisio K.A., Xiao L. (2005). Distribution of *Cryptosporidium* Genotypes in storm event water samples from three watersheds in New York. Appl. Environ. Microbiol..

[210] Feng Y., Alderisio K.A., Yang W., Blancero L.A., Kuhne W.G., Nadareski C.A., Reid M., Xiao L. (2007). *Cryptosporidium* Genotypes in Wildlife from a New York Watershed. Appl. Environ. Microbiol..

[211] Robinson G., Wright S., Elwin K., Hadfield S.J., Katzer F., Bartley P.M., Hunter P.R., Nath M., Innes E.A., Chalmers R.M. (2010). Redescription of *Cryptosporidium cuniculus* Inman and Takeuchi, 1979 (Apicomplexa: Cryptosporidiidae): Morphology, biology and phylogeny. Int. J. Parasitol..

[212] Hadfield S.J., Chalmers R.M. (2012). Detection and characterization of *Cryptosporidium cuniculus* by real-time PCR. Parasitol. Res..

[213] Naguib D., Roellig D., Arafat N., Xiao L. (2021). Genetic Characterization of *Cryptosporidium cuniculus* from Rabbits in Egypt. Pathogens.

[214] Koehler A.V., Rashid M.H., Zhang Y., Vaughan J.L., Gasser R.B., Jabbar A. (2018). First cross-sectional, molecular epidemiological survey of *Cryptosporidium*, *Giardia* and *Enterocytozoon* in alpaca (*Vicugna pacos*) in Australia. Parasites Vectors.

[215] Kaupke A., Kwit E., Chalmers R., Michalski M., Rzeżutka A. (2014). An outbreak of massive mortality among farm rabbits associated with *Cryptosporidium* infection. Res. Vet. Sci..

[216] Liu X., Zhou X., Zhong Z., Chen W., Deng J., Niu L., Wang Q., Peng G. (2014). New Subtype of *Cryptosporidium cuniculus* Isolated from Rabbits by Sequencing the Gp60 Gene. J. Parasitol..

[217] Zhang X., Qi M., Jing B., Yu F., Wu Y., Chang Y., Zhao A., Wei Z., Dong H., Zhang L. (2016). Molecular Characterization of *Cryptosporidium* spp., *Giardia duodenalis*, and *Enterocytozoon bieneusi* in Rabbits in Xinjiang, China. J. Eukaryot. Microbiol..

[218] Zhang W., Shen Y., Wang R., Liu A., Ling H., Li Y., Cao J., Zhang X., Shu J., Zhang L. (2012). *Cryptosporidium cuniculus* and *Giardia duodenalis* in Rabbits: Genetic Diversity and Possible Zoonotic Transmission. PLoS ONE.

[219] Yang Z., Yang F., Wang J., Cao J., Zhao W., Gong B., Yan J., Zhang W., Liu A., Shen Y. (2018). Multilocus sequence typing and population genetic structure of *Cryptosporidium cuniculus* in rabbits in Heilongjiang Province, China. Infect. Genet. Evol..

[220] Kváč M., Hofmannová L., Hlásková L., Květoňová D., Vítovec J., McEvoy J., Sak B. (2010). *Cryptosporidium erinacei* n. sp. (Apicomplexa: Cryptosporidiidae) in hedgehogs. Vet. Parasitol..

[221] Laatamna A.E., Wagnerová P., Sak B., Květoňová D., Aissi M., Rost M., Kváč M. (2013). Equine cryptosporidial infection associated with *Cryptosporidium* hedgehog genotype in Algeria. Vet. Parasitol..

[222] Dyachenko V., Kuhnert Y., Schmaeschke R., Etzold M., Pantchev N., Daugschies A. (2010). Occurrence and molecular characterization of *Cryptosporidium* spp. genotypes in European hedgehogs (*Erinaceus europaeus* L.) in Germany. Parasitology.

[223] Hofmannová L., Hauptman K., Huclová K., Květoňová D., Sak B., Kváč M. (2016). *Cryptosporidium erinacei* and *C*. parvum in a group of overwintering hedgehogs. Eur. J. Protistol..

[224] Prediger J., Horčičková M., Hofmannová L., Sak B., Ferrari N., Mazzamuto M.V., Romeo C., Wauters L.A., McEvoy J., Kváč M. (2017). Native and introduced squirrels in Italy host different *Cryptosporidium* spp.. Eur. J. Protistol..

[225] Power M.L., Cheung-Kwok-Sang C., Slade M., Williamson S. (2009). *Cryptosporidium fayeri*: Diversity within the GP60 locus of isolates from different marsupial hosts. Exp. Parasitol..

[226] Takaki Y., Takami Y., Watanabe T., Nakaya T., Murakoshi F. (2020). Molecular identification of *Cryptosporidium* isolates from ill exotic pet animals in Japan including a new subtype in *Cryptosporidium fayeri*. Vet. Parasitol. Reg. Stud. Rep..

[227] Qian W., Zhang Y., Jiang Y., Zhao A., Lv C., Qi M. (2020). Molecular characterization of *Cryptosporidium* spp. in minks (*Neovison vison*), blue foxes (*Vulpes lagopus*), and raccoon dogs (*Nyctereutes procyonoides*) in farms from Xinjiang, Northwest China. Parasitol. Res..

[228] Feng Y., Lal A.A., Li N., Xiao L. (2011). Subtypes of *Cryptosporidium* spp. in mice and other small mammals. Exp. Parasitol..

[229] Dettwiler I., Troell K., Robinson G., Chalmers R.M., Basso W., Rentería-Solís Z.M., Daugschies A., Mühlethaler K., Dale M.I., Raghavendra J.B. (2021). TIDE Analysis of *Cryptosporidium* Infections by gp60 Typing Reveals Obscured Mixed Infections. J. Infect. Dis..

[230] Yanta C.A., Bessonov K., Robinson G., Troell K., Guy R.A. (2021). CryptoGenotyper: A new bioinformatics tool for rapid *Cryptosporidium* identification. Food Waterborne Parasitol..

[231] Braima K., Zahedi A., Egan S., Austen J., Xiao L., Feng Y., Witham B., Pingault N., Perera S., Oskam C. (2021). Molecular analysis of cryptosporidiosis cases in Western Australia in 2019 and 2020 supports the occurrence of two swimming pool associated outbreaks and reveals the emergence of a rare C. hominis IbA12G3 subtype. Infect. Genet. Evol..

[232] Vinayak S., Pawlowic M.C., Sateriale A., Brooks C.F., Studstill C.J., Bar-Peled Y., Cipriano M.J., Striepen B. (2015). Genetic modification of the diarrhoeal pathogen *Cryptosporidium parvum*. Nature.

[233] Vangah S.J., Katalani C., Booneh H.A., Hajizade A., Sijercic A., Ahmadian G. (2020). CRISPR-Based Diagnosis of Infectious and Noninfectious Diseases. Biol. Proced. Online.

[234] Yu F., Zhang K., Wang Y., Li D., Cui Z., Huang J., Zhang S., Li X., Zhang L. (2021). CRISPR/Cas12a-based on-site diagnostics of *Cryptosporidium parvum* IId-subtype-family from human and cattle fecal samples. Parasites Vectors.

[235] Fan Y., Feng Y., Xiao L. (2019). Comparative genomics: How has it advanced our knowledge of cryptosporidiosis epidemiology?. Parasitol. Res..

[236] Baptista R., Cooper G., Kissinger J. (2021). Challenges for *Cryptosporidium* Population Studies. Genes.

[237] Xu Z., Li N., Guo Y., Feng Y., Xiao L. (2020). Comparative genomic analysis of three intestinal species reveals reductions in secreted pathogenesis determinants in bovine-specific and non-pathogenic *Cryptosporidium* species. Microb. Genom..

[238] Su J., Jin C., Wu H., Fei J., Li N., Guo Y., Feng Y., Xiao L. (2019). Differential expression of three *Cryptosporidium* species-specific MEDLE Proteins. Front. Microbiol..

[239] Guo Y., Tang K., Rowe L.A., Li N., Roellig D.M., Knipe K., Frace M., Yang C., Feng Y., Xiao L. (2015). Comparative genomic analysis reveals occurrence of genetic recombination in virulent *Cryptosporidium hominis* subtypes and telomeric gene duplications in *Cryptosporidium parvum*. BMC Genom..

[240] Pallen M.J. (2014). Diagnostic metagenomics: Potential applications to bacterial, viral and parasitic infections. Parasitology.

[241] Lopes R.J., Merida A.M., Carneiro M. (2017). Unleashing the potential of public genomic resources to find parasite genetic data. Trends Parasitol..

[242] Beghini F., Pasolli E., Truong T.D., Putignani L., Cacciò S.M., Segata N. (2017). Large-scale comparative metagenomics of Blastocystis, a common member of the human gut microbiome. ISME J..

[243] Franssen F.F.J., Janse I., Janssen D., Caccio S.M., Vatta P., van der Giessen J.W.B., van Passel M.W.J. (2021). Mining public metagenomes for environmental surveillance of parasites: A proof of principle. Front. Microbiol..

